# “Trauma to the Eye”—A Low Fidelity Resident Teaching Module for Identifying and Treating a Retrobulbar Hematoma

**DOI:** 10.15766/mep_2374-8265.11075

**Published:** 2021-01-25

**Authors:** Jared Raikin, Ronald V. Hall, Dimitrios Papanagnou

**Affiliations:** 1 Medical Student, Sidney Kimmel Medical School, Thomas Jefferson University; 2 Associate Professor, Department of Emergency Medicine, Thomas Jefferson University; 3 Associate Dean of Faculty Development, Department of Emergency Medicine, Thomas Jefferson University

**Keywords:** Retrobulbar Hematoma, Simulation, Low Fidelity, Lateral Canthotomy and Cantholysis, Just-in-Time Training, Emergency Medicine, Ophthalmology, Case-Based Learning, Clinical/Procedural Skills Training

## Abstract

**Introduction:**

A retrobulbar hematoma (RH) is a serious time-dependent diagnosis due to its potential for permanent damage of the optic nerve, resulting in blindness. Emergency medicine (EM) physicians face the challenge of recognizing this time-sensitive injury and treating it before irreversible damage occurs. Due to its relative infrequency in the emergency department, residents may not have adequate experience in recognizing and treating RH.

**Methods:**

This educational intervention outlined a simulated scenario that we developed to educate EM residents to diagnose RH and perform an emergent lateral canthotomy and cantholysis (LCC). Participating residents were asked to obtain a history and perform a physical examination that was consistent with a 34-year-old patient presenting with pushing behind the eye suggesting RH. Once residents made a diagnosis, they practiced performing an emergent LCC on a low-fidelity task trainer supplemented with a novel checklist. The residents completed an assessment questionnaire before and after the teaching module to measure the educational intervention's effectiveness.

**Results:**

Learners' scores significantly improved in the ability to recognize and treat RH (12%, *p* < .001), in confidence in performing the procedure (18%, *p* < .001), but did not significantly decrease in stress (−10%, *p* = .058). The intervention was effective in improving preparedness, with all participants indicating that they felt more prepared to treat RH compared to before the educational intervention.

**Discussion:**

This educational intervention is a successful resource that can decrease cases of preventable blindness by improving EM residents' ability to recognize and treat RHs.

## Educational Objectives

By the end of this activity, learners will be able to:

1. Diagnose a retrobulbar hematoma (RH).
2. Measure intraocular pressures.
3. Identify relevant anatomical structures of the eye.
4. Perform lateral canthotomy and cantholysis confidently.
5. Describe appropriate follow-up care for RH.

## Introduction

A traumatic injury to the eye represents a unique challenge to physicians as they could result in poor outcomes if not treated in a timely fashion. An emergency medicine (EM) resident's ability to assess and treat these injuries within a couple hours of their occurrence can prevent permanent disabilities. Training and experience with these injuries can be invaluable in helping prepare EM residents improve their patients' outcomes. Retrobulbar hemotomas (RH) are one type of traumatic injury to the eye for which further training of residents would be beneficial.

RH is described as trauma to the head causing blood to fill the cavity behind the eye. If not promptly identified and treated, this buildup of blood can compress the optic nerve and result in permanent blindness. Since other injuries present similarly to RH, it is important to be able to distinguish them due to varying treatments.^1^ Following prompt recognition of the RH, a lateral canthotomy and cantholysis (LCC) is indicated for treatment. Crushing and cutting of the lateral canthus and evacuating the blood in the potential space behind the eye within 2 hours of the trauma relieves pressure from the optic nerve by giving the eye space to expand. When done properly, these procedures can prevent potential blindness and return vision to the injured eye.^2^

Previous studies have shown that there is a clear need for additional educational efforts in teaching the proper performance of LCC. In a study done in an American level-one trauma center, there averaged only five lateral canthotomies per year.^3^ Due to their minimal frequency, residents do not have sufficient opportunity to observe and practice this procedure in a clinical setting. The same study also found that while incidents of RH are an uncommon occurrence, up to 48% of cases result in blindness. Another study found that 83% of British equivalents of second-year residents and below were not competent in properly diagnosing and treating RH.^4^ These studies suggested that many of these cases of blindness are preventable if they are treated properly within 2 hours of injury. This indicates a need for furthering education in the recognition of RH and performance of LCC.

The current simulation standard for teaching procedures in a nonclinical scenario is replicating the injury on a cadaver and performing the relevant procedures. This higher-fidelity simulation replicates the relevant anatomy and provides a real feel of tissue, allowing for more optimal learning conditions. An educational intervention using cadaver simulation for EM residents described the important steps taken in an LCC cadaver simulation.^5^ While higher-fidelity cadaver simulations have clear benefits, they are also expensive and provide limited hands-on practice opportunities for learners.

This problem was highlighted by an intervention that felt the need to create a low-fidelity simulation alternative for performing an LCC in resource-limited areas in Africa.^6^ This low-fidelity alternative is cheaper, more easily replicable, and allows for more attempts compared to the high-fidelity cadaver model. While there are many advantages to this low-fidelity module, only the participants' subjective comfort with the procedure was measured. Furthermore, there were no educational materials to supplement these cadaver and low-fidelity models. Our simulation module has the opportunity to be an important supplementary resource for training learners to recognize and treat RH.

There exists a clear gap in the education curriculum for EM resident identification of RH and performance of an LCC. While new low-fidelity modules have been created to address these needs, they lack supplementation with clinical scenarios, checklists, and quantitative analysis to measure their efficacy. This educational intervention quantitatively measured the success of the low-fidelity module paired with a clinical scenario and checklist, using a novel assessment questionnaire. It was predicted that EM residents who participated in the educational curriculum plus the low-fidelity RH training would show improvement in the knowledge domain, affective domain, as well as feel more prepared to perform an LCC.

This educational intervention supplemented by a low-fidelity module is important because it can provide residents with increased training in promptly recognizing RH and properly treating the injury with an LCC. A literature review of *MedEdPORTAL* and other peer-reviewed journals showed that this is the first curriculum that quantitatively studied and supported the effectiveness of an education intervention supplemented by a low-fidelity RH simulation module for EM. This curriculum could in turn help future patients avoid preventable blindness.

## Methods

### Development

This curriculum was developed because of residents' poor performance in recognition and treatment of RH. Its purpose was to fill in a gap in the education on treatment for RHs by giving learners an opportunity to identify the trauma and practice performing the treatment. EM residents received this curriculum during their ear, nose, and throat (ENT)/ophthalmology block as part of a trauma educational training to recognize and treat various injuries. The facilitator should be familiar with the recognition and treatment for RH and the participants should be competent in basic diagnostic skills expected of a resident.

### Equipment/Environment

All items used in this module were cost effective and easily obtained at any sport store and local drug store. The eye module used in this simulation was the exact same module made using detailed instructions in the paper by Kong;^6^ the models were constructed using their model (Appendix A). Two copies of the assessment questionnaire (Appendix B) and a copy of the novel checklist (Appendix C) were printed for each participant. Audiovisual equipment was used to present the PowerPoint, which included the case and supplemental images (Appendix D). Procedural equipment included the following items:

• 5% Betadine solution
• Hemostat or needle drivers
• Forceps
• Iris or suture scissors
• Tonopen
• Lidocane with epinephrine
• 25 gauge 5/8 needles
• Syringe

### Personnel

Only one person/facilitator was needed for this case. They were responsible for playing the patient by answering questions about the patient and providing insight to facilitate learning for the participants by asking thought-provoking questions.

### Implementation

EM residents were handed a pretest assessment questionnaire (Appendix B) and given 10 minutes to complete it once they arrived to the simulation in a classroom. Once completed, we presented the simulation case, which was made to replicate a real scenario that described a patient who suffered trauma to the eye from a direct blow to the orbital globe with a squash ball. The facilitator, as the patient, described to the residents that they presented to the ED and that there was pain, swelling, and bruising around the left eye. Resident participants went through an oral history and physical exam, which was given by the facilitator, to gather pertinent information and form a differential diagnosis. The facilitator gave information about the patient according to the case presentation (Appendix E). During this time, gaps in learners' knowledge were identified and supplemented with information provided in Appendix E. The case ended when the learners reached the diagnosis of RH. The facilitator handed out the eye models and checklists (Appendix C) and began the debrief session.

### Assessment

The questionnaire we made (Appendix B) measured the knowledge domain of participants' ability to answer factual questions in recognizing and treating an RH. The affective domain was measured with questions using a 4-point rating scale (1 = *no confidence/stress*, 4 = *extreme confidence/stress*), and participants were asked to indicate their confidence performing the procedure alone and level of anticipated stress levels if performing the procedure alone. Higher scores indicated more confidence and stress. This was done to create a spectrum by which learners could express their feelings, which could later be quantified. Using a 3-point rating scale, participants were asked on the posttest only if they felt more, equally, or less prepared to perform the procedure compared to how they felt before the module.

The questionnaire was made based on key steps found in the literature for recognition and treatment of an RH. The content was reviewed by an EM attending physician who populated alternative answers based on clinical knowledge. In the module, participants received a novel checklist to supplement their learning. Various educational resources were used to compile the novel assessment questionnaire and checklist.^7–9^

### Debriefing

Our recommended method for learner debriefing focused around simulation and opportunity to practice the LCC indicated in this case. The debriefing process included a review of the case with supplementary images and data (Appendix D), walking through the checklist (Appendix C), and deliberate practice performing a LCC using the eye model. It was important to emphasize to participants what will happen if an RH is left untreated, the importance of proper follow-up care, and the proper use of a Tonopen.

## Results

Thirty-one EM resident physicians participated in our simulation module that was facilitated by an EM attending. Creation of the eye modules was the majority of the preparation that was required for this simulation. All 31 residents completed both the pre- and posttest. On the knowledge domain portion of the questionnaire, participants' pretest scores averaged 82% with an improvement in their posttest scores that averaged 94%. In regards to the affective domain portion of the questionnaire, participants had an average pretest score of 56% and posttest score of 73% for their level of confidence in performing the procedure alone, indicating an improvement. Participants had an average pretest score of 54% and posttest score of 44% for their level of stress if performing the procedure alone indicating a decrease in stress.

A paired Student's *t* test was used on the R software platform to interpret the data. Participants' scores improved on the assessment questionnaire in the knowledge (12%, *p* < .001) and affective domain (confidence: 18%, *p* < .001; stress: −10%, *p* = .058; Table). The posttest questionnaire found that 100% of participants (31 of 31) felt more prepared to perform the procedure relative to before the module. The participants were asked if they would be interested in participating in a refresher course in the future and 94% (29 of 31) indicated *yes* or *maybe*.

**Table.**
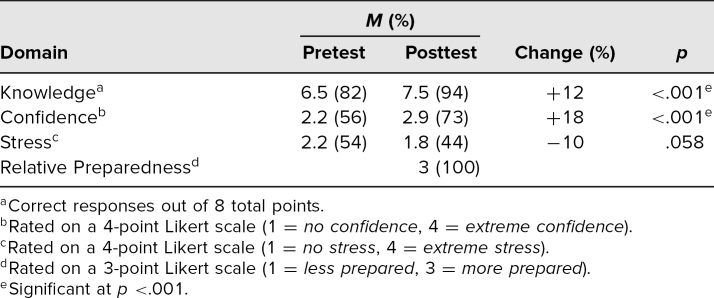
Assessment Questionnaire of Knowledge and Affective Domain Before and After Lateral Canthotomy and Cantholysis Simulation (*N* = 31)

## Discussion

To address the high rates of preventable blindness due to RH, we have created a collection of resources including checklists, didactic content, and assessment tools, and supplemented them with an already established inexpensive, accessible, and easily replicable low-fidelity model^6^ with the intention of teaching residents how to recognize RH and properly perform an LCC. Through our evaluation of this curriculum it has been supported that learners had significantly improved knowledge and increased confidence. All participants indicated that they felt more prepared to treat RH after the module.

The results demonstrate that this novel educational curriculum supplemented with a low-fidelity module was effective in meeting the stated learning objectives of teaching EM residents how to properly diagnose an RH, measure intraocular pressure, identify relevant anatomy, and perform an LCC. This was supported by the data showing an improvement in both the knowledge and affective domain scores from the pretest to posttest questionnaire. The results suggested that this curriculum significantly improved their ability to recognize and treat an RH, their confidence in performing the procedure, and their feelings of relative preparedness, but did not significantly decrease their stress.

This curriculum was a successful educational resource that can potentially decrease cases of preventable blindness and improve outcomes by improving EM residents' ability to recognize and treat RHs. Our assessment of this educational intervention supported that this curriculum was unique in that it can be used as a comprehensive collection of educational resources to supplement simulation tools used to teach identification and treatment of RH. This is the first publication that quantitatively studied and supported the effectiveness of a curriculum using a low-fidelity RH simulation module for EM residents. This teaching module can now be used for any EM residency program looking to reinforce the skills needed to recognize and care for a patient suffering from RH including practicing the proper steps of performing an LCC.

Several areas of change have been addressed to improve this education intervention from what the authors have learned through its implementation. More images have been added to the case PowerPoint to provide visual cues for this injury, improve clarity of relevant anatomy, and provide an example of how to perform an LCC. Similarly, more supplemental images were added to the case to show exactly what steps needed to be taken to perform an LCC. This will help decrease confusion among the learners and allow them to see how to perform an LCC properly before attempting it on the models. It was also found to be important to dedicate extra time to demonstrate how to properly use a Tonopen due to challenges that the learners faced. Lastly, the 4-point scale to measure learners' confidence and stress was kept because it allowed for a spectrum that allowed for quantification of affect. The *maybe* option in the question addressing learners' interest in a refresher course was removed to provide a clearer result.

While the results clearly indicated the success of this curriculum and low-fidelity module, it was limited in that it was not performed in direct comparison to the currently used cadaver models. Therefore, it cannot be assumed that this curriculum and low-fidelity module was superior or equivalent to the current teaching practice. Instead we can conclude that this curriculum can be used as an effective supplement to all existing teaching methods. Similarly, despite support for improvement in the knowledge and affective domain, participants' performance on the actual procedure was not assessed. This measurement was outside the scope of this educational intervention and therefore we cannot conclude that this module will directly improve outcomes of LCCs. Further studies should be performed to assess the patient outcomes of residents who participated in this educational module to see if there is improvement in patient outcomes.

The results of the educational intervention also suggested that this curriculum does not directly decrease the stress EM residents would expect if asked to perform this procedure alone. While minimizing stress would be ideal, we predict that only through actual experience performing this procedure multiple times would we see a decrease in the stress anticipated with performing an LCC alone. Another limitation of this intervention was the lack of information of how this knowledge will decay over time. This was outside the scope of this educational intervention but would be interesting to explore in the future by repeating the assessment at various intervals after the educational intervention. Through this intervention we have also identified a gap in the research for a peer-reviewed validated checklist in treating RH.

This curriculum has potential use outside of resident education in the classroom. We believe that our novel checklists supplemented with low-fidelity eye models are well suited for just-in-time training when residents are faced with an RH in a clinical setting. These checklists and models can be kept around the ED, and when a patient with RH presents to the ER they can be used as an educational resource to walk through the appropriate steps they need to take. This style of teaching can be expanded to any injury with the appropriate simulation materials. As more weaknesses are identified in residents' ability to properly treat uncommon injuries, more curricula combined with low-fidelity models can be created as a way to help fill in those gaps. Curricula supplemented by low-fidelity simulation have endless possibilities and will continue to have a significant role in medical education. We believe that our results supported that this curriculum combined with the low-fidelity module can lead to improved patient outcomes and fewer cases of preventable blindness due to RHs.

## Appendices

Model Construction.docxAssessment Questionnaire.docxRH Checklist.docxCase and Supplemental Images.pptxSimulation Case Template.docx
All appendices are peer reviewed as integral parts of the Original Publication.
