# Supplementary material for: “Trauma to the Eye”—A Low Fidelity Resident Teaching Module for Identifying and Treating a Retrobulbar Hematoma
Source: MedEdPORTAL. 2021 Jan 25;17:11075. doi: 10.15766/mep_2374-8265.11075 (PMC7837065; doi:10.15766/mep_2374-8265.11075)
Supplement: Supplementary file 1 — Model Construction.docxAssessment Questionnaire.docxRH Checklist.docxCase and Supplemental Images.pptxSimulation Case Template.docx [file mep_2374-8265.11075-s001.zip › A. Model Construction.docx]

Appendix A. Model Construction

The model below is the exact same model created by Kong (2018)

The following items are needed in order to construct the low fidelity model used in this case:

Standard size Ping-Pong

10ml Ziploc recyclable container

Pressure Foam Tape

Transpore tape 3 M^TM^

Tape Role (or something of similar height)

Rubber Band

Scissors

Scalpel

Marker

Model Assembly:

Step 1- cut out a rectangular shape out of the bottom of the Ziploc container at the indentation lines (Figure 1)

Step 2- Cut the rubber band in half, then lay it flat and cut the middle third lengthwise to simulate the two canthal tendons.

Step 3- Use two pieces of Transpore tape and fold each over the upper and lower bisected rubber band leaving overlap to allow for later sticking (Figure 2)

Step 4- Cut 2 pieces of foam tape, and create two small flaps laterally and fold them down leaving you with one large middle flap and two small outer flaps.

(Figure 3)

Step 5- Place the foam tape at the upper and lower parts of the rubber band and then fold the large middle flap over the rubber band. (Figure 4)

Step 6- Plate the Transpore tape over the corners of the outer Ziploc container and secure the foam tape on the opposite corners. (Figure 5)

Step 7- Secure the model with Transpore tape around the outer edges of the model to prevent it from unsticking.

Step 8- Use marker to color Ping-Pong ball to look like an eye, then place it on an empty tape roll into the Tupperware container and secure it with the lid.

(Figure 6)

Figure 1: Figure 2:


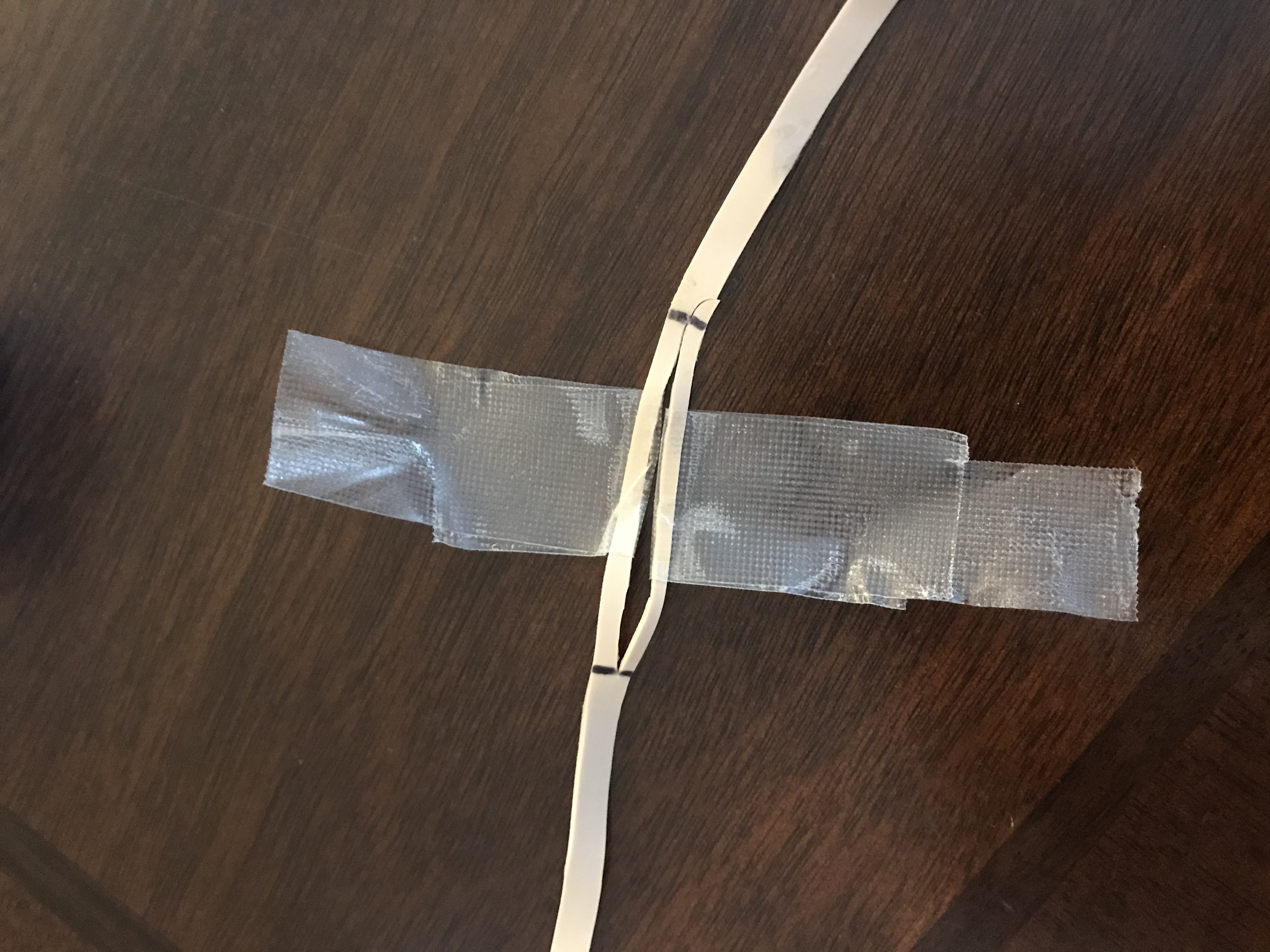

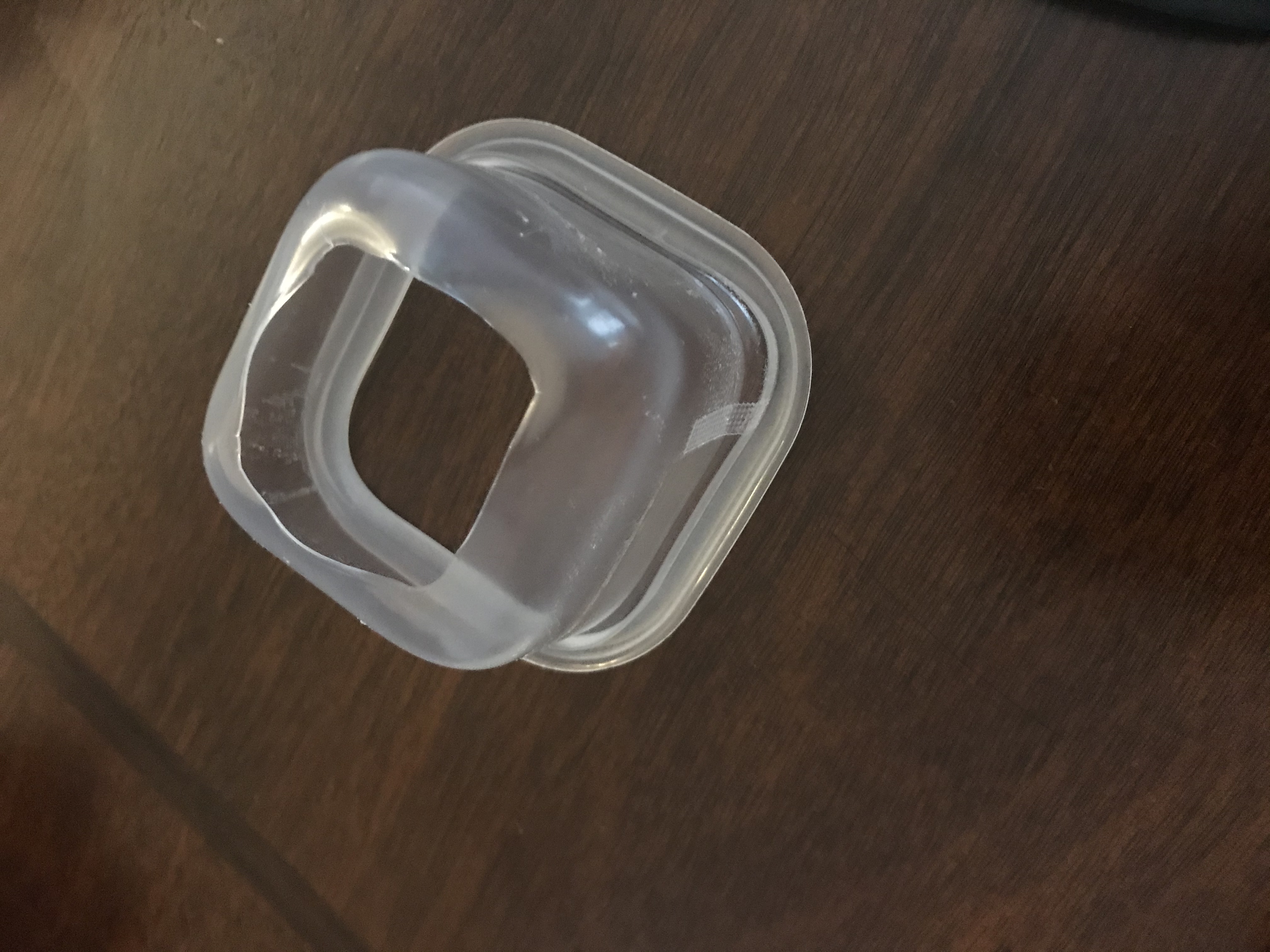


(Author owned image)

(Author owned image)

Figure 3: Figure 4:


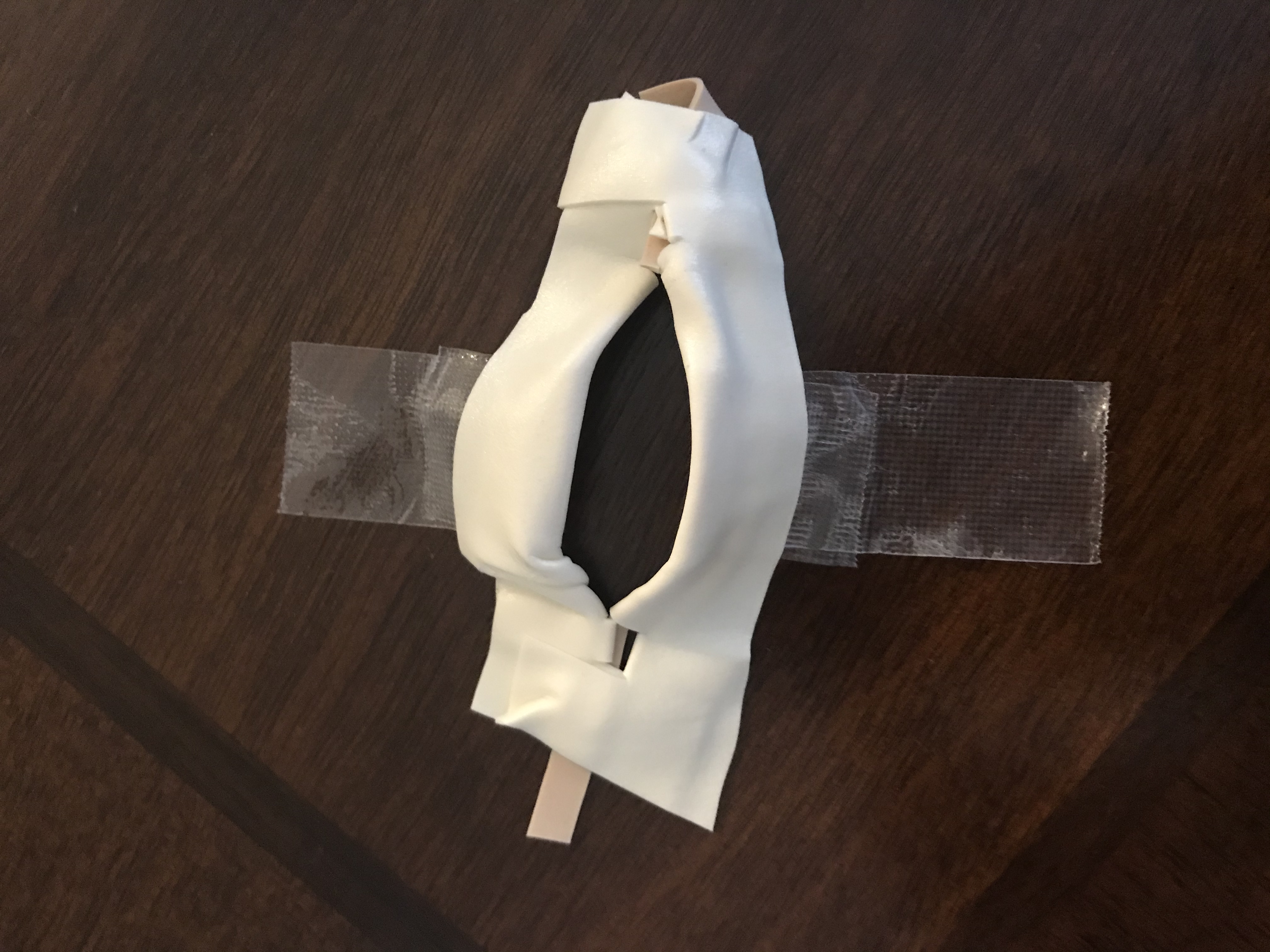


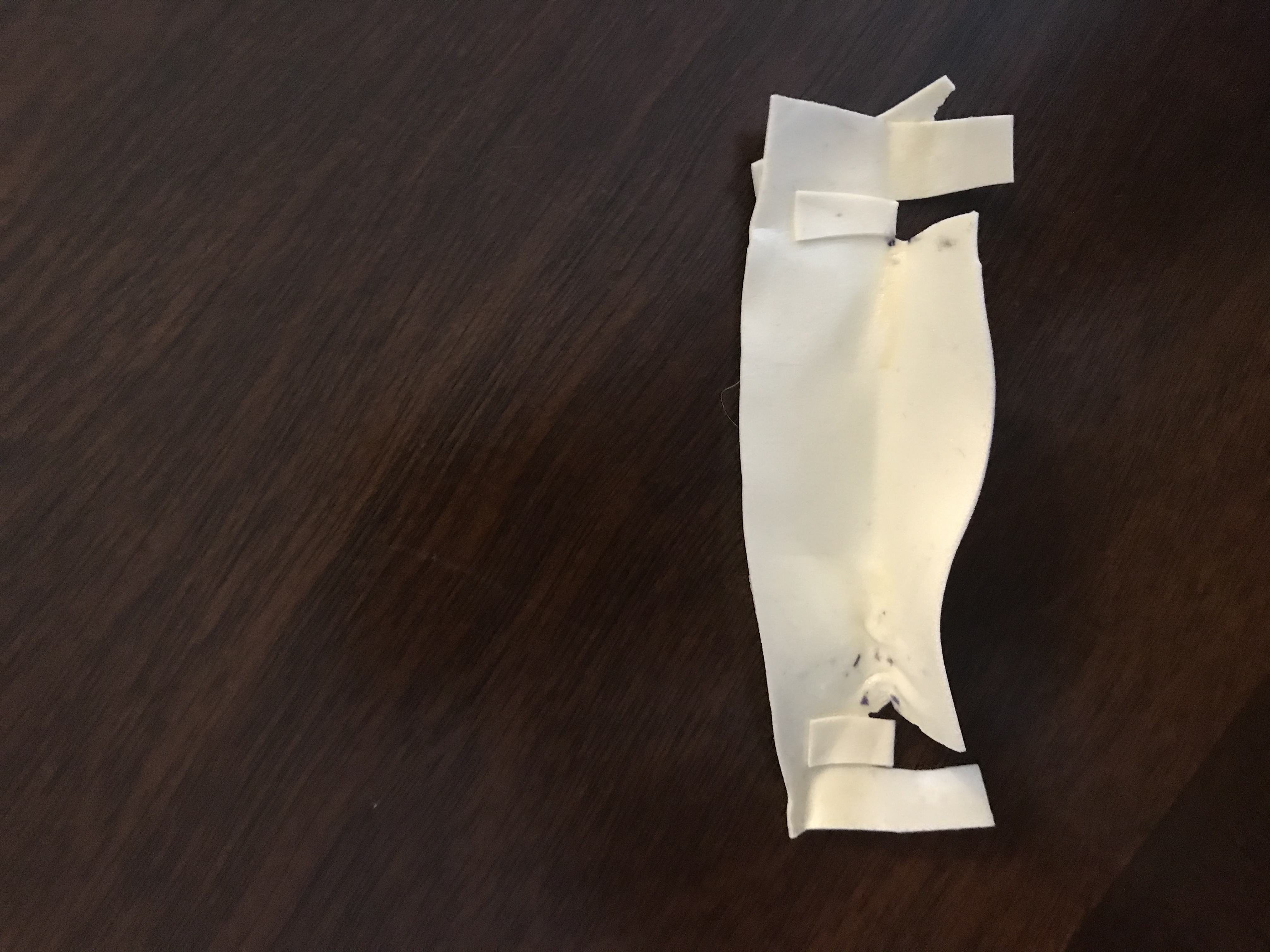


(Author owned image)

(Author owned image)

Figure 5: Figure 6:


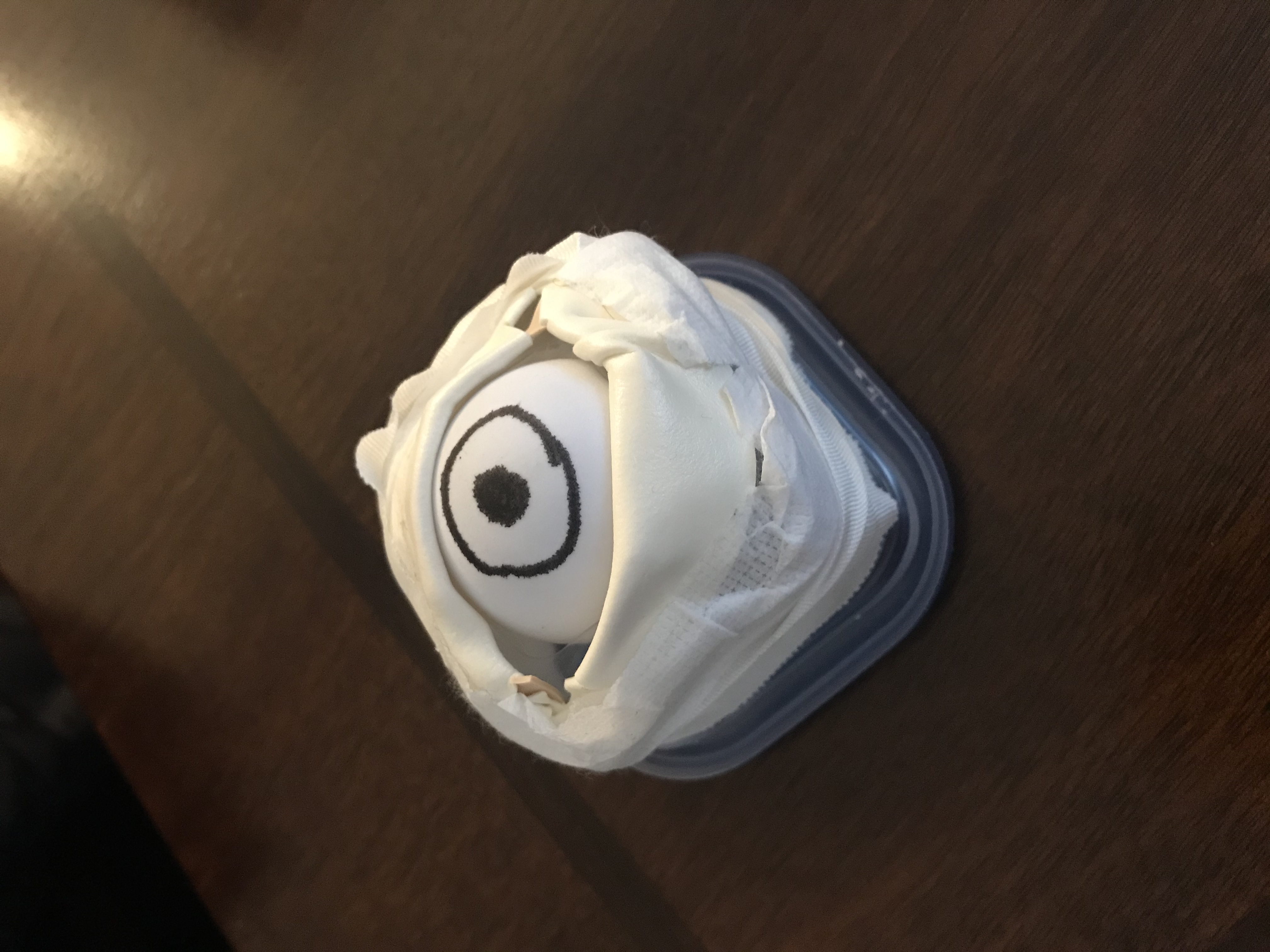


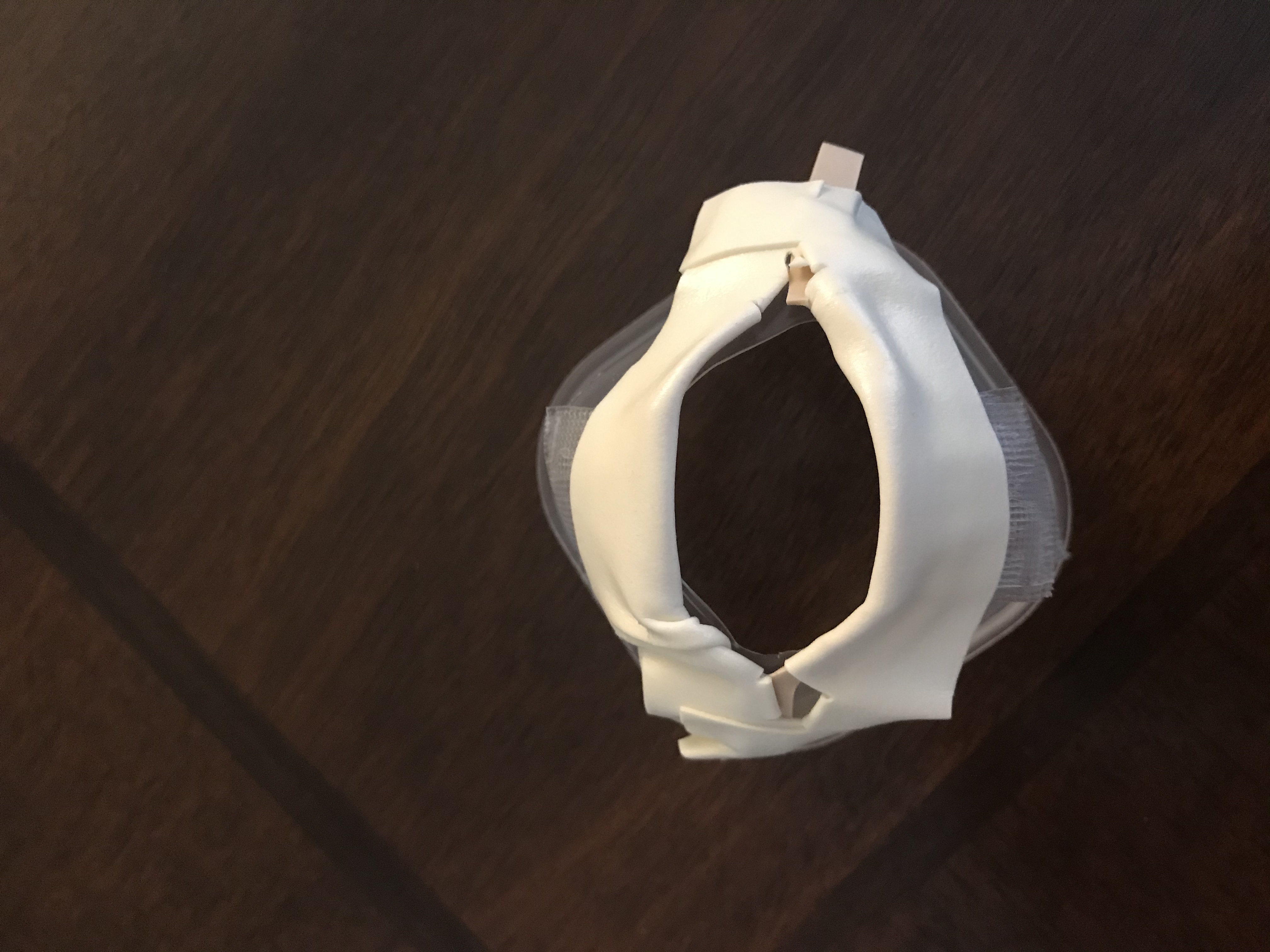


(Author owned image)

(Author owned image)

1. Kong R, Kaya DP, Cioe-Pena E, Greenstein J. A low fidelity eye model for lateral canthotomy training. *Afr J Emerg Med.* 2018;8(3):118-122.
