# Supplementary material for: “Trauma to the Eye”—A Low Fidelity Resident Teaching Module for Identifying and Treating a Retrobulbar Hematoma
Source: MedEdPORTAL. 2021 Jan 25;17:11075. doi: 10.15766/mep_2374-8265.11075 (PMC7837065; doi:10.15766/mep_2374-8265.11075)
Supplement: Supplementary file 1 — Model Construction.docxAssessment Questionnaire.docxRH Checklist.docxCase and Supplemental Images.pptxSimulation Case Template.docx [file mep_2374-8265.11075-s001.zip › B. Assessment Questionnaire.docx]

**Appendix B. Assessment Questionnaire for Lateral Canthotomy and Cantholysis:**

**(Please circle the best answer)**

1. Which of the following findings on physical examination necessitate performing an emergent lateral canthotomy and cantholysis?

1. **Decreased visual acuity, Intraocular pressure of 50mmHg, Proptosis**
2. Decreased visual acuity, hyphema, pain of out proportion to the physical exam
3. Decreased visual acuity, diplopia, intraocular pressure of 20 mmHg
4. Decreased visual acuity, hyphema, Siedel’s sign
5. Decreased visual acuity, hyphema, diplopia

2. You are evaluating a patient complaining of right eye pain from a direct blow with a softball. There is marked soft tissue edema and the eye feels firm upon palpation. As part of your evaluation, you attempt to measure her intraocular pressures. What is the pressure range that indicated a lateral canthotomy and cantholysis?

1. >10 mmHg
2. >20 mmHg
3. >30 mmHg
4. **>40 mmHg**
5. >50 mmHg

3. What is a contraindication for a lateral canthotomy and cantholysis?

1. Anticoagulation
2. **Globe Rupture**
3. Multiple facial trauma
4. Raccoon eyes
5. Third Nerve Palsy

4. What is the most likely complication if a lateral canthotomy and cantholysis is not preformed when indicated?

1. Acute Angle Closure Glaucoma
2. **Blindness due to compression of the ocular nerve**
3. Blindness due to compression of the retina
4. Disfigurement of the eyelid
5. Third nerve palsy

5. What are the follow up recommendations for a patient who had a LCC?

1. **Admit for observation to ensure reduction in intraocular pressure**
2. Admit to ophthalmology for immediate surgical repair
3. Discharge after observation in the ED with eye patch and antibiotics
4. Discharge to home with timolol eye drop and ophthalmology consult
5. Immediate discharge to home with follow at ophthalmology clinic

6. Which structures need to be cut in a lateral canthotomy?

1. **Lateral canthus and inferior crus of lateral canthal tendon**
2. Lateral canthus and superior crus of lateral canthal tendon
3. Lateral canthus and lateral canthus tendon
4. Lateral canthus, lateral canthal tendon, and lateral rectus muscle
5. Lateral canthus and lateral rectus muscle

7. How long must you clamp the lateral canthus before cutting?

1. 20 seconds
2. 30 seconds
3. 40 seconds
4. 50 seconds
5. **60 seconds**

8. Which of the following steps is most crucial after completion of the procedure?

1. Administration of timolol to prevent conversion to angle closure glaucoma
2. Eye shield for protection
3. Mannitol to ensure intraocular pressures remain stable
4. Repeat orbital CT
5. **Tonopen measurement to confirm reduction in pressure**

How confident would you be performing this procedure by yourself?

A. Extremely confident B. Moderately confident C. Mildly confident D. Not confident

How stressed would you be performing this procedure by yourself?

A. Not Stressed B. Mildly Stressed C. Moderately Stressed D. Extremely Stressed

*Post-test Questions:

How prepared do you feel to preform this procedure compared to before this module?

A. More prepared B. Less Prepared C. Equally as Prepared

Would you be interested in participating in a refresher course in the future?

A. No B. Yes
