# Supplementary material for: “Trauma to the Eye”—A Low Fidelity Resident Teaching Module for Identifying and Treating a Retrobulbar Hematoma
Source: MedEdPORTAL. 2021 Jan 25;17:11075. doi: 10.15766/mep_2374-8265.11075 (PMC7837065; doi:10.15766/mep_2374-8265.11075)
Supplement: Supplementary file 1 — Model Construction.docxAssessment Questionnaire.docxRH Checklist.docxCase and Supplemental Images.pptxSimulation Case Template.docx [file mep_2374-8265.11075-s001.zip › C. RH Checklist.docx]

**Appendix C. RH Checklist for Lateral Canthotomy and Cantholysis:**

| **Preparation** | |
| --- | --- |
| Gather appropriate tools | - 5% Betadine solution - Lidocaine with Epinephrine - Syringe with 25 gauge 5/8 needle - Hemostat or needle driver - Iris or suture scissors - Forceps - Tonopen |
| Page Ophthalmology |  |
| Visual acuity | - Document using Snellen chart |
| Prepare surgical sight | - Clear away debris saline - Clean skin with Betadine 5% solution |
| Check for Globe Rupture | - Apply fluorescein stain to eye and observe for Seidel sign - If stain ‘runs out’, it indicates globe rupture - If stain doesn’t ‘run out’, must rule out posterior globe rupture - Must still decompress if ruptured globe present with RH |
| Numb Cornea | - Use proparacaine eye drops |
| Assess IOP | - Use Tonopen to measure Intraorbital Pressure (IOP) - If >40mmHg then procedure indicated |

| **Lateral Canthomy and Cantholysis** | |
| --- | --- |
| Numb surgical site | - Inject local anesthetic for numbing in lateral canthus   - Inject from lateral canthus to orbital rim   - Direct needle tip away from globe   - Should be 1-2cc |
| Relieve pressure behind eye | - Crush Lateral Canthus with hemostat or needle driver to control bleeding - Should be done for 1 minute - Cut through all layers of tissue along lateral canthus with iris scissors - Should be 1-2cm in length laterally - Apply tension on lower lid with forceps to identify lateral canthal tendon - Tendon is just inferior and posterior to lateral canthal fold - Cut through lateral canthal ligament with iris scissors at inferior crus - Cut should be perpendicular to first cut - You will feel lower lid give way if done properly - Measure intraocular Pressure (IOP) with Tonopen to confirm pressure released - If still >40mmHg then decompression is inadequate - Lift upper lid and cut superior crus if pressure still >40mmHg - Should release the rest of the pressure |

| **Follow Up** | |
| --- | --- |
| Assess eye function | - Measure eye response to light and movement - Response should be seen within 15 minutes of pressure release - Measure visual acuity with Snellen chart |
| Post surgical care | - Admit patient for observation to ensure full reduction in IOP - Apply eye shield until follow up - Schedule Ophthalmology follow up for patient |

**Sources:**

1. Justin Morgenstern, "Procedure: Lateral Canthotomy", First10EM blog, April 1, 2015. Available at: <https://first10em.com/lateral-canthotomy/>.

2. Linden JA, Renner GS. Trauma to the globe. Emerg Med Clin North Am 1995;13(3):581-605.

3. Larsen M, Wieslander S. Acute orbital compartment syndrome after lateral blow-out fracture effectively relieved by lateral cantholysis. Acta Ophthalmol Scand 1999;77:232-3.

4. UptoDate
