# Supplementary material for: “Trauma to the Eye”—A Low Fidelity Resident Teaching Module for Identifying and Treating a Retrobulbar Hematoma
Source: MedEdPORTAL. 2021 Jan 25;17:11075. doi: 10.15766/mep_2374-8265.11075 (PMC7837065; doi:10.15766/mep_2374-8265.11075)
Supplement: Supplementary file 1 — Model Construction.docxAssessment Questionnaire.docxRH Checklist.docxCase and Supplemental Images.pptxSimulation Case Template.docx [file mep_2374-8265.11075-s001.zip › D. Case and Supplemental Images.pptx]

## Slide 1
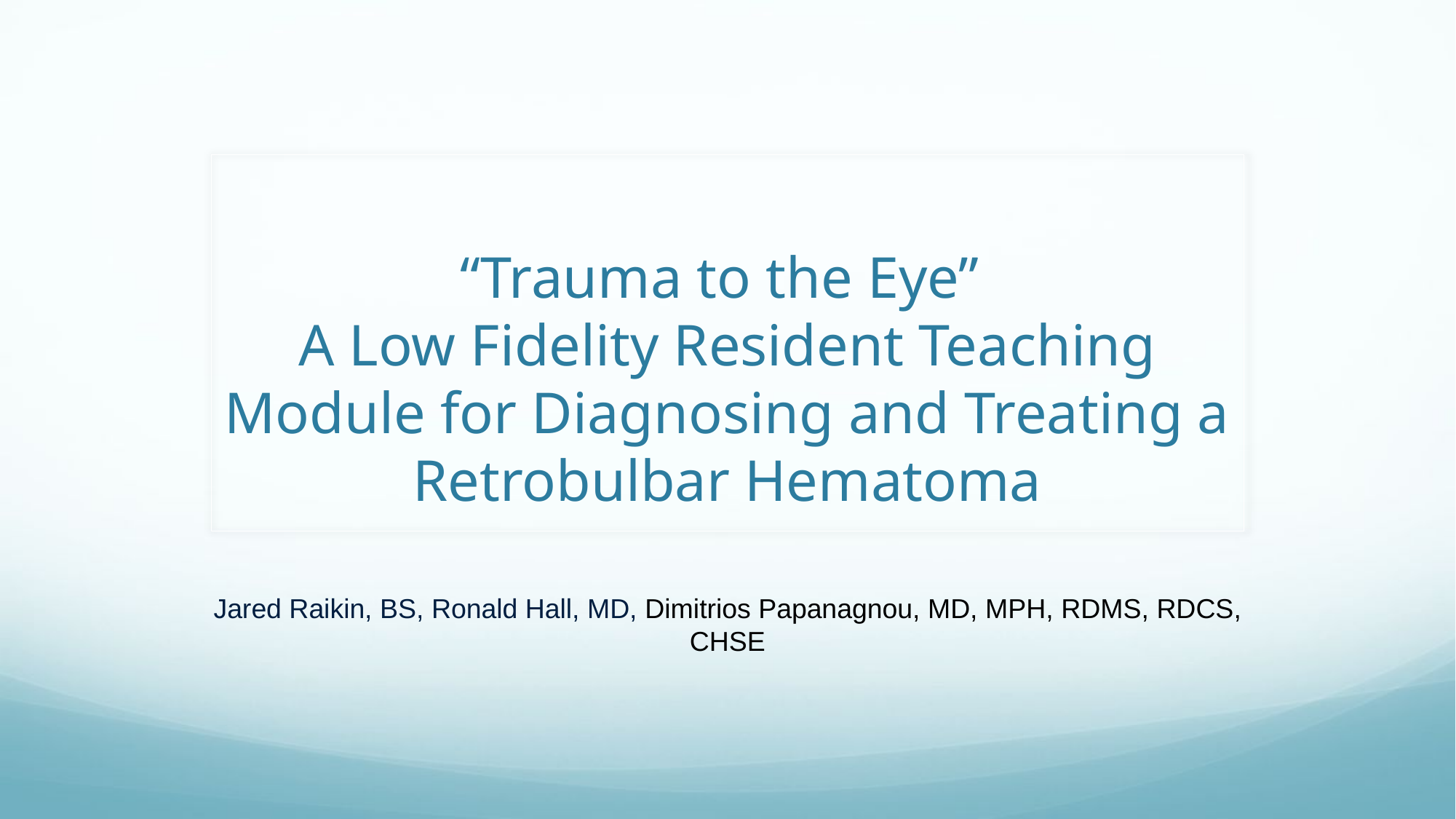

# “Trauma to the Eye” A Low Fidelity Resident Teaching Module for Diagnosing and Treating a Retrobulbar Hematoma
Jared Raikin, BS, Ronald Hall, MD, Dimitrios Papanagnou, MD, MPH, RDMS, RDCS, CHSE

## Slide 2
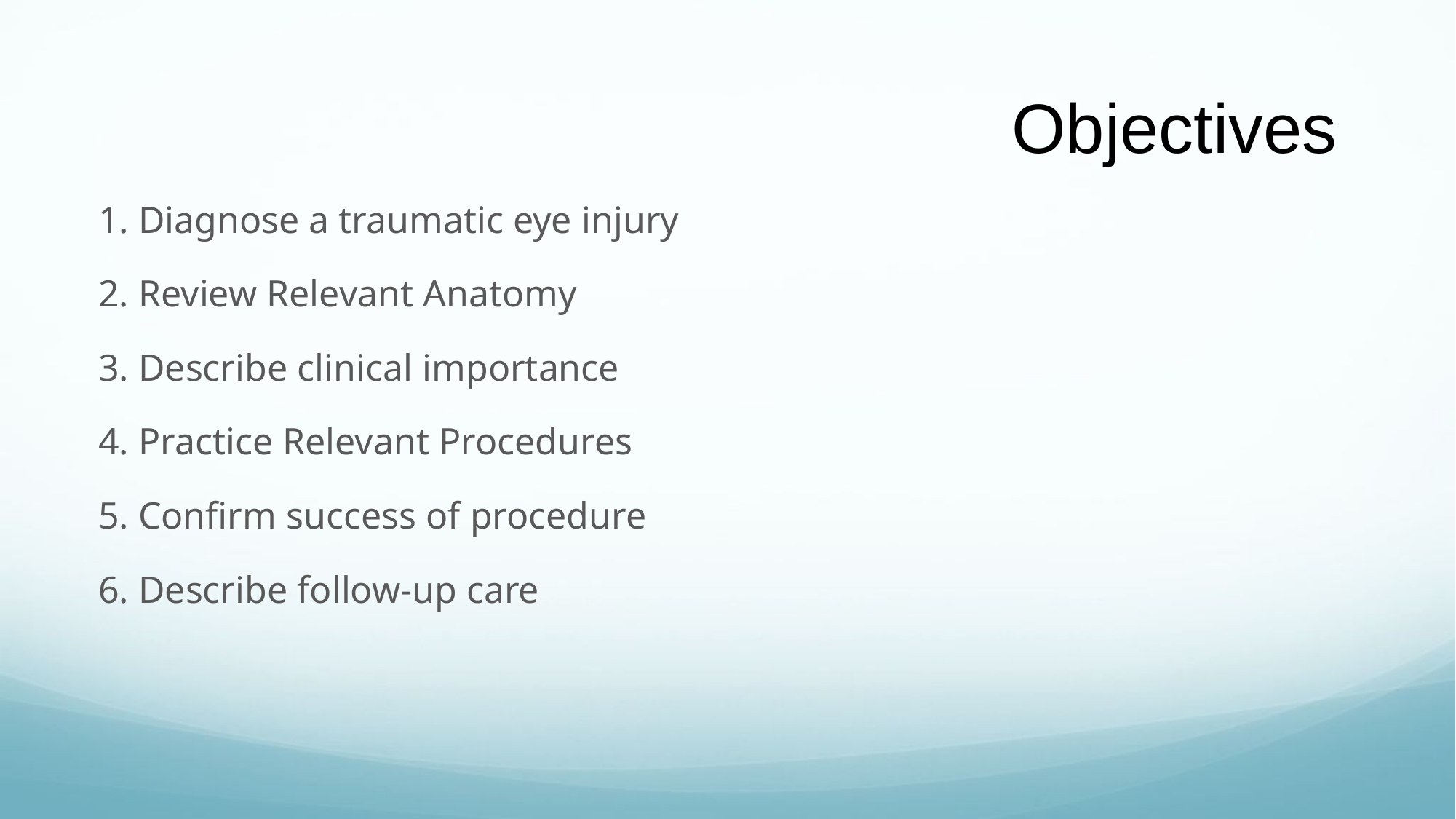

# Objectives
1. Diagnose a traumatic eye injury
2. Review Relevant Anatomy
3. Describe clinical importance
4. Practice Relevant Procedures
5. Confirm success of procedure
6. Describe follow-up care

## Slide 3
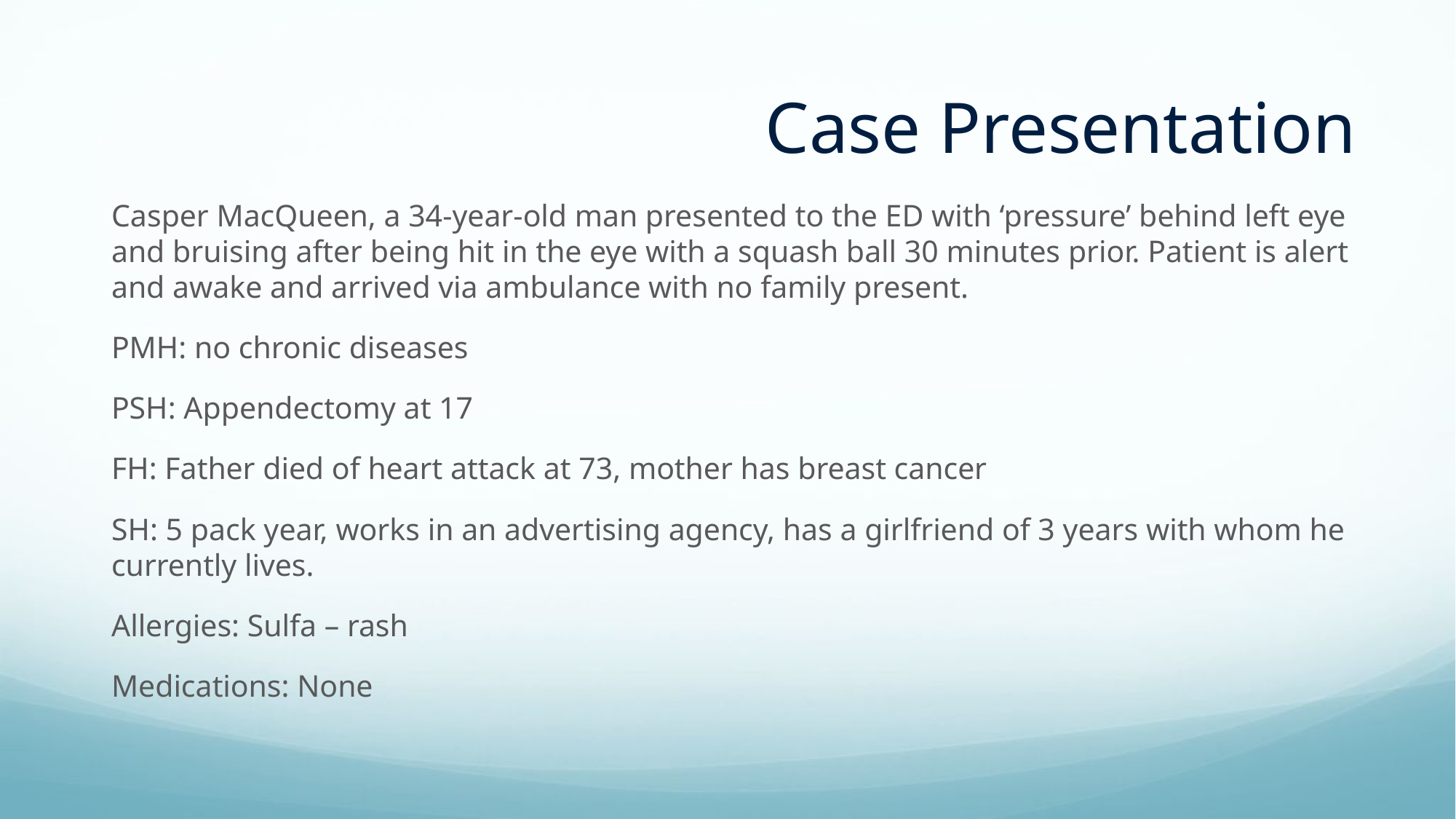

# Case Presentation
Casper MacQueen, a 34-year-old man presented to the ED with ‘pressure’ behind left eye and bruising after being hit in the eye with a squash ball 30 minutes prior. Patient is alert and awake and arrived via ambulance with no family present.
PMH: no chronic diseases
PSH: Appendectomy at 17
FH: Father died of heart attack at 73, mother has breast cancer
SH: 5 pack year, works in an advertising agency, has a girlfriend of 3 years with whom he currently lives.
Allergies: Sulfa – rash
Medications: None

## Slide 4
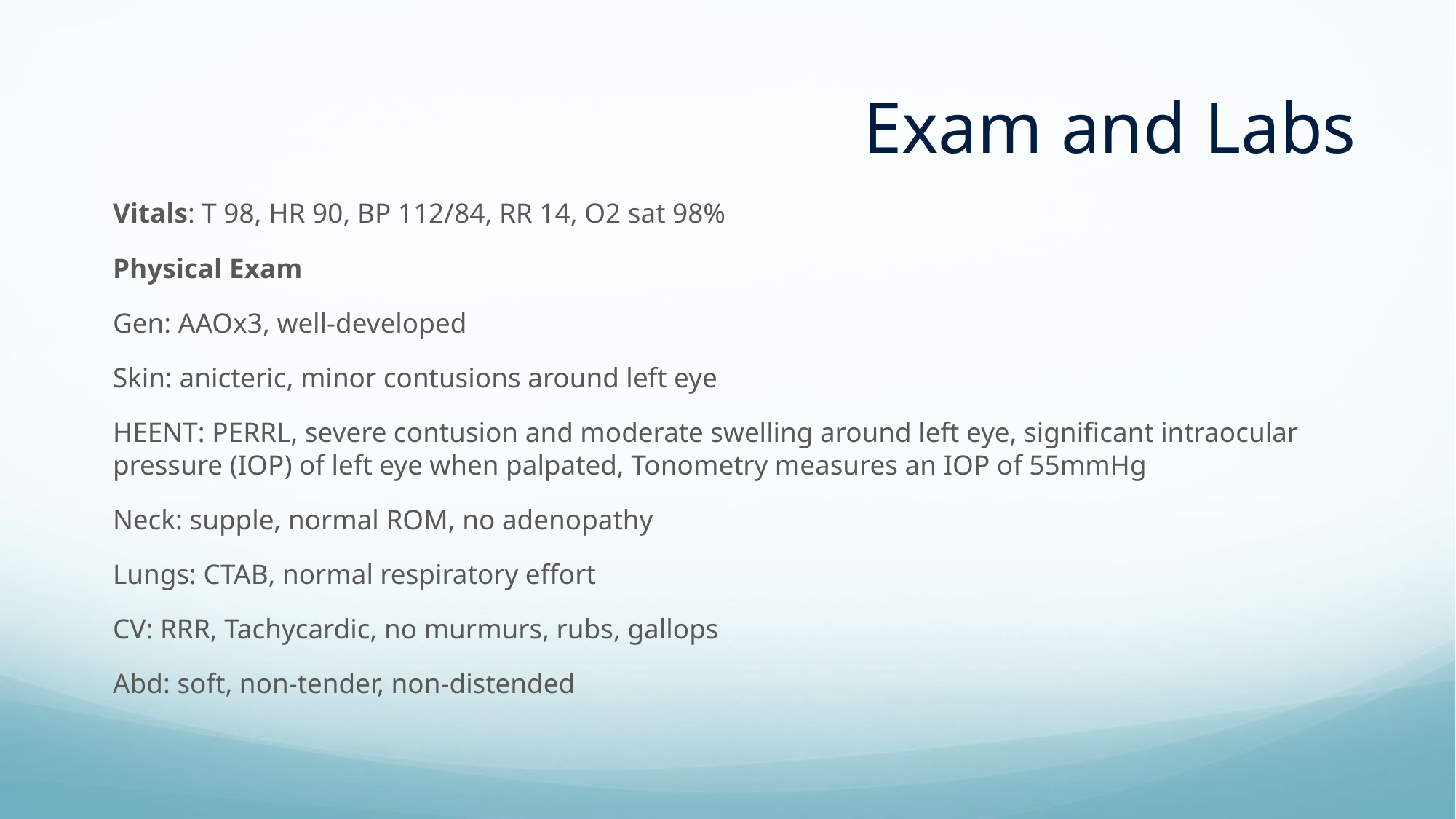

# Exam and Labs
Vitals: T 98, HR 90, BP 112/84, RR 14, O2 sat 98%
Physical Exam
Gen: AAOx3, well-developed
Skin: anicteric, minor contusions around left eye
HEENT: PERRL, severe contusion and moderate swelling around left eye, significant intraocular pressure (IOP) of left eye when palpated, Tonometry measures an IOP of 55mmHg
Neck: supple, normal ROM, no adenopathy
Lungs: CTAB, normal respiratory effort
CV: RRR, Tachycardic, no murmurs, rubs, gallops
Abd: soft, non-tender, non-distended

## Slide 5
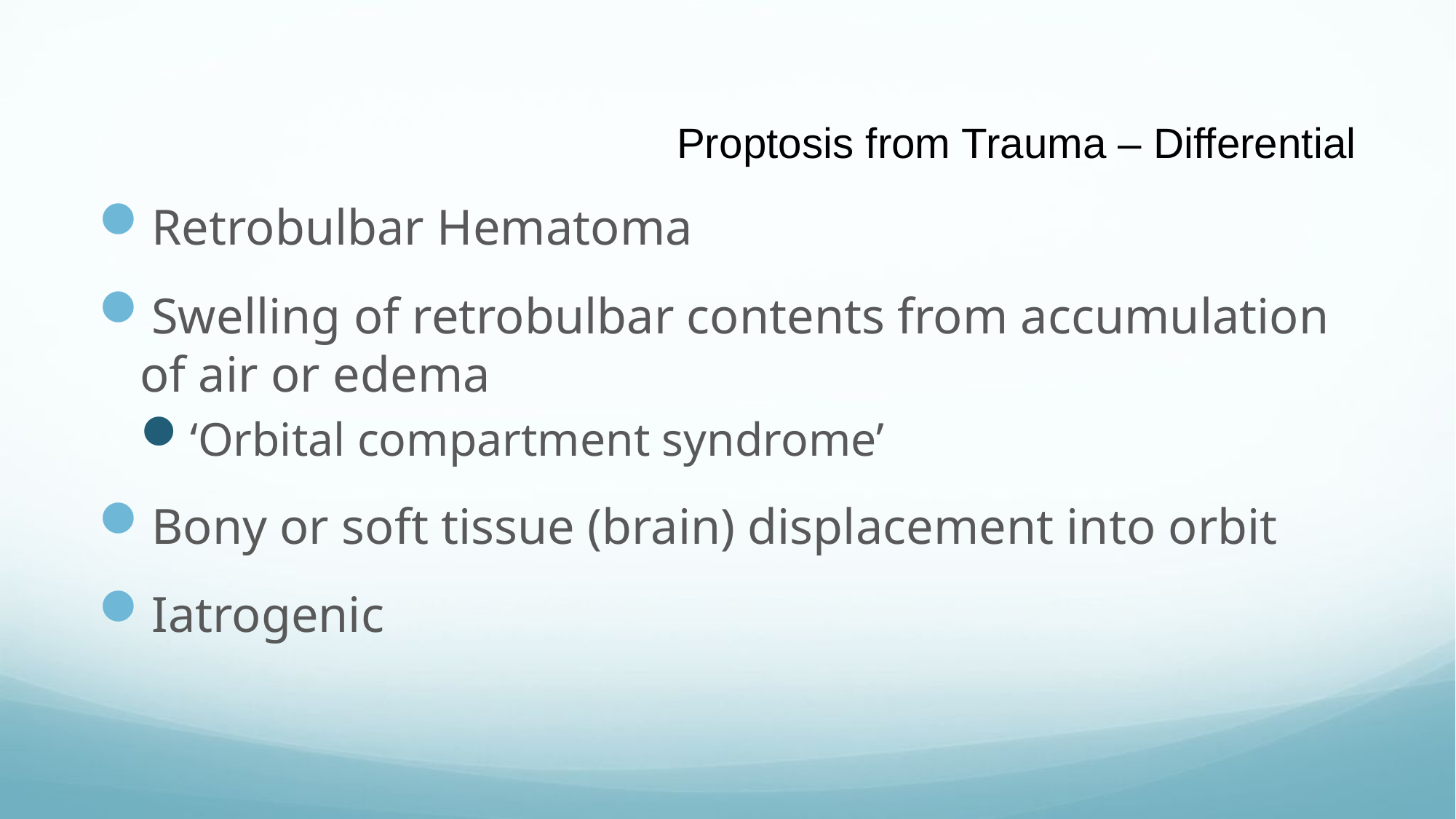

# Proptosis from Trauma – Differential
Retrobulbar Hematoma
Swelling of retrobulbar contents from accumulation of air or edema
‘Orbital compartment syndrome’
Bony or soft tissue (brain) displacement into orbit
Iatrogenic

## Slide 6
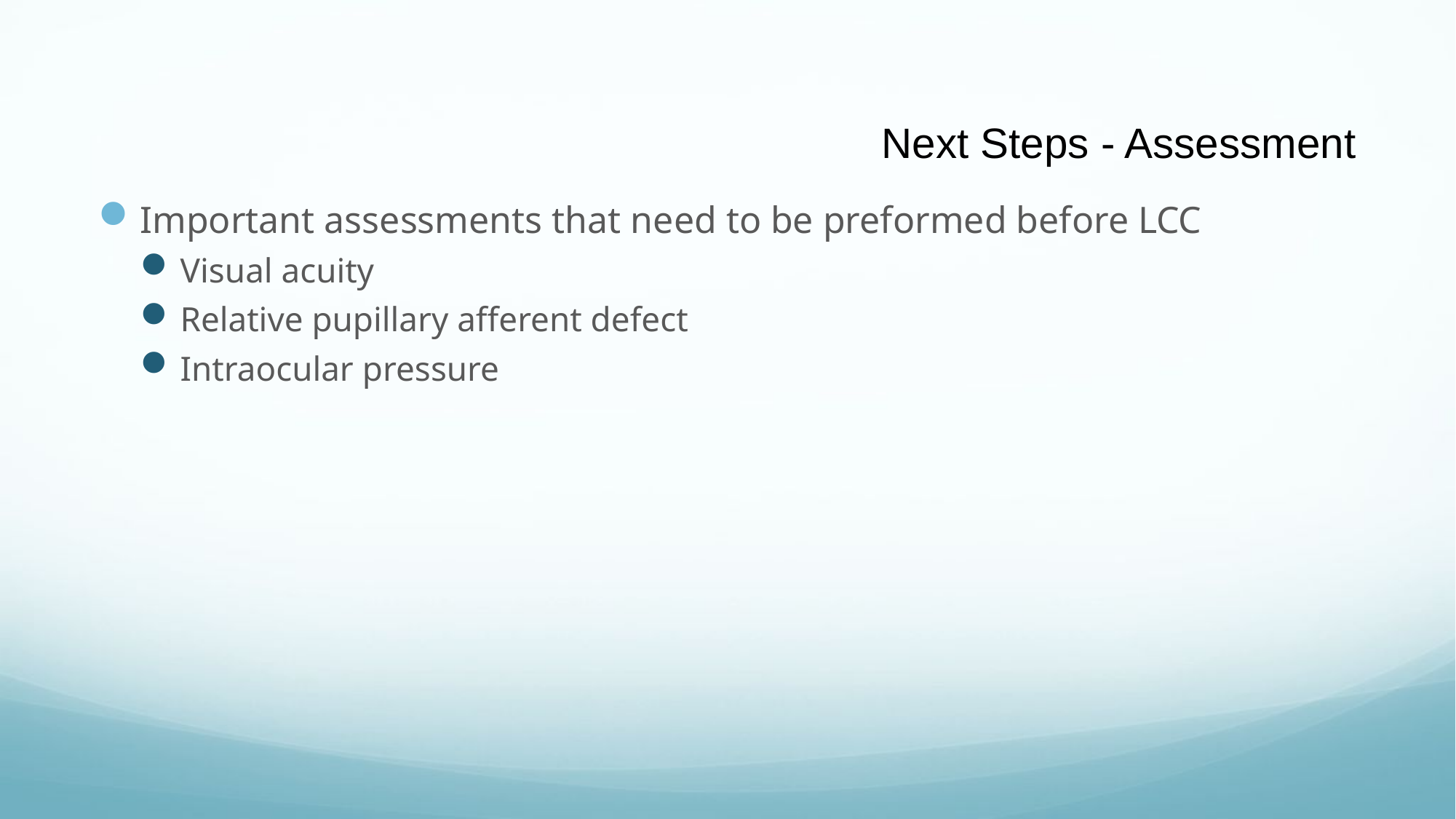

# Next Steps - Assessment
Important assessments that need to be preformed before LCC
Visual acuity
Relative pupillary afferent defect
Intraocular pressure

## Slide 7
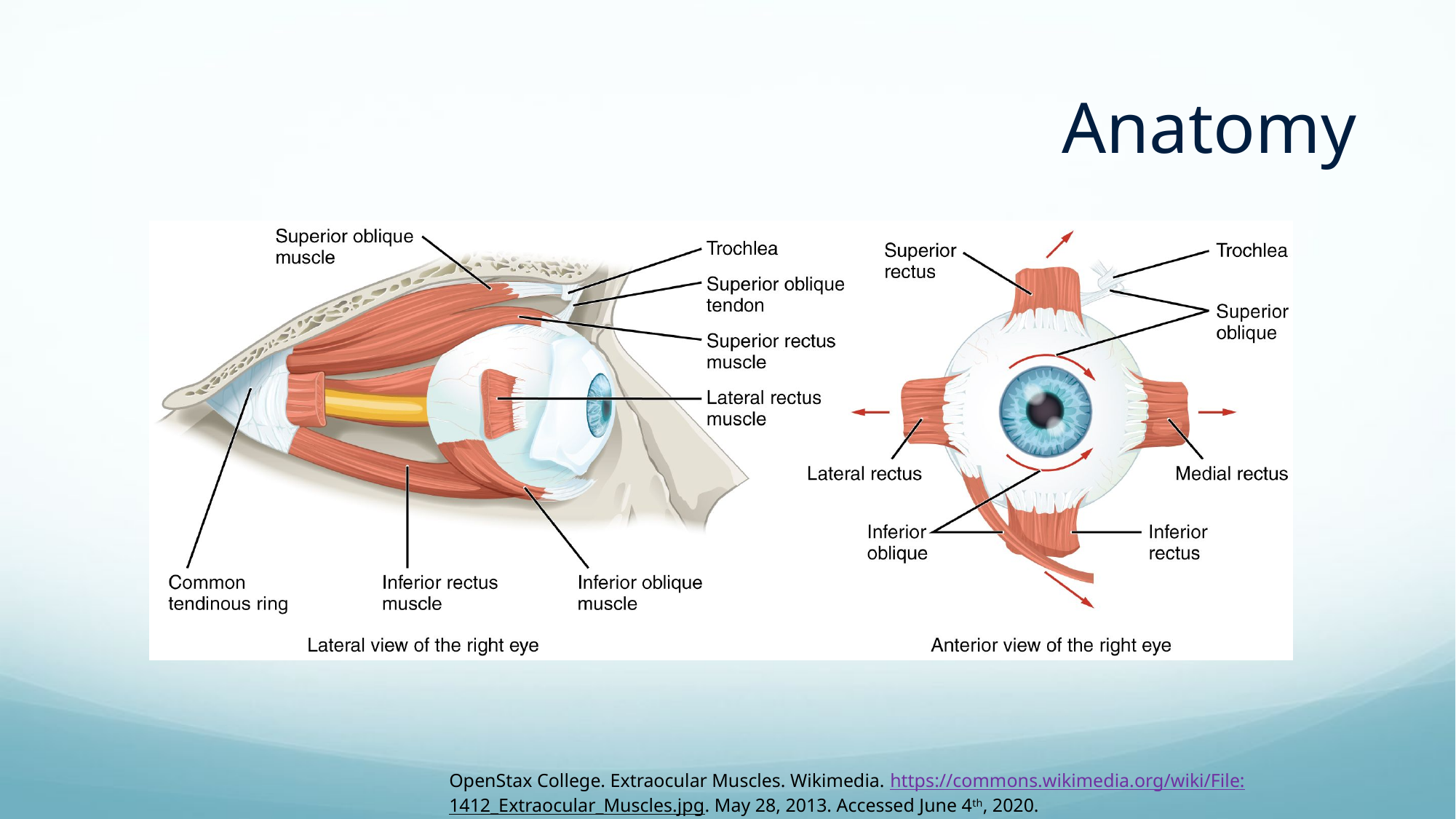

# Anatomy
OpenStax College. Extraocular Muscles. Wikimedia. https://commons.wikimedia.org/wiki/File:1412_Extraocular_Muscles.jpg. May 28, 2013. Accessed June 4th, 2020.

## Slide 8
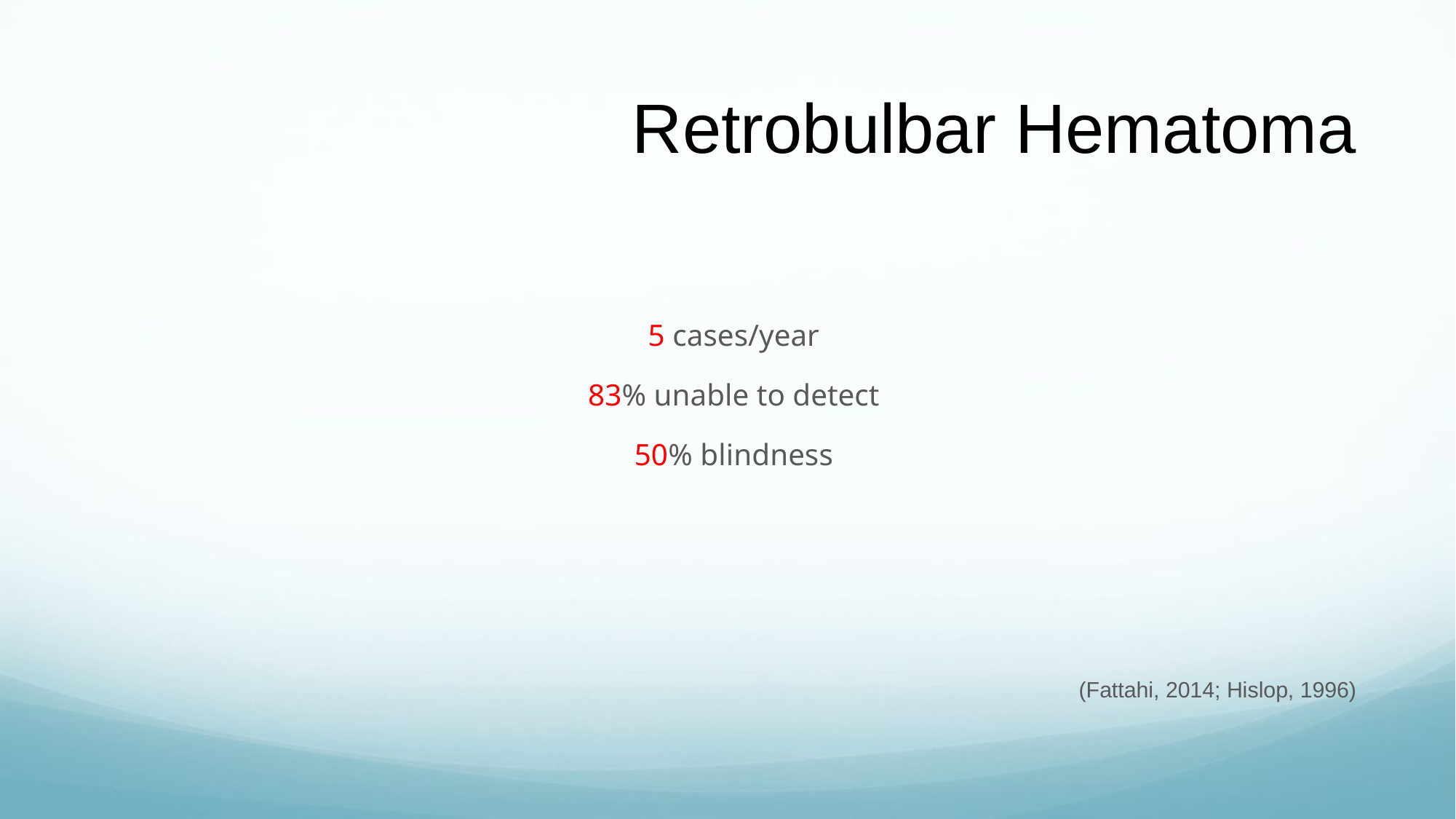

# Retrobulbar Hematoma
5 cases/year
83% unable to detect
50% blindness
(Fattahi, 2014; Hislop, 1996)

## Slide 9
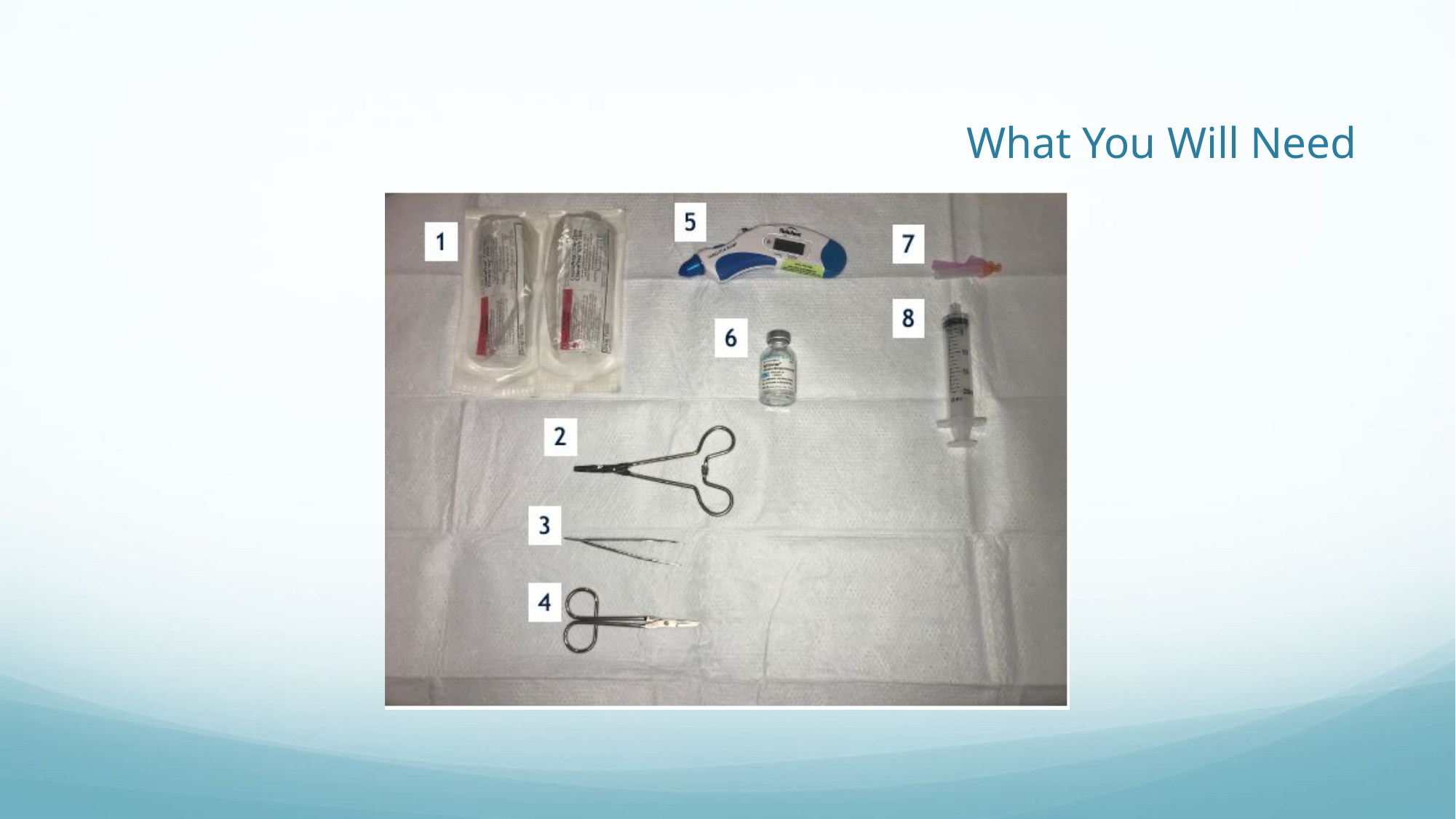

# What You Will Need

## Slide 10
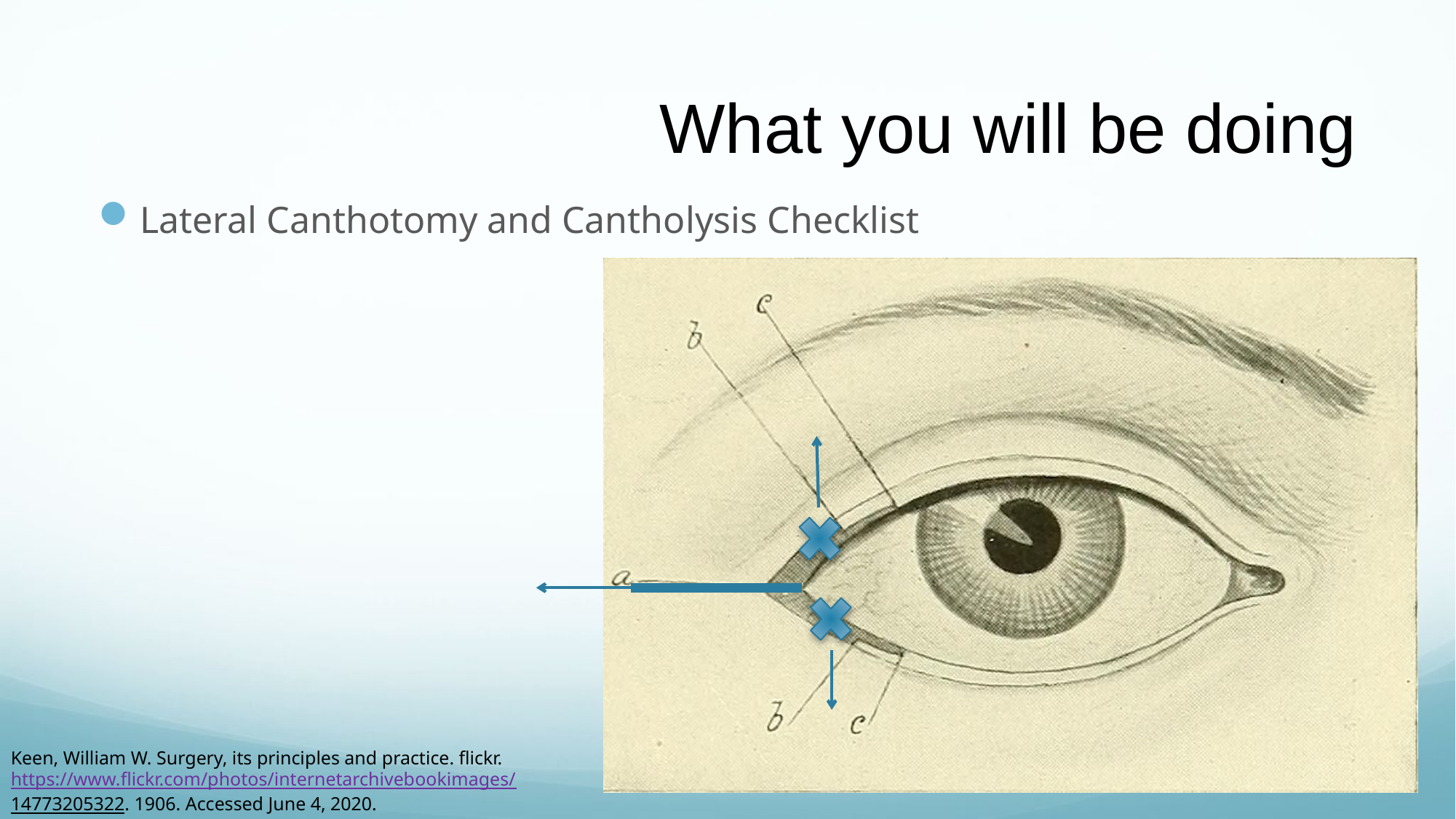

# What you will be doing
Lateral Canthotomy and Cantholysis Checklist
Keen, William W. Surgery, its principles and practice. flickr. https://www.flickr.com/photos/internetarchivebookimages/14773205322. 1906. Accessed June 4, 2020.

## Slide 11
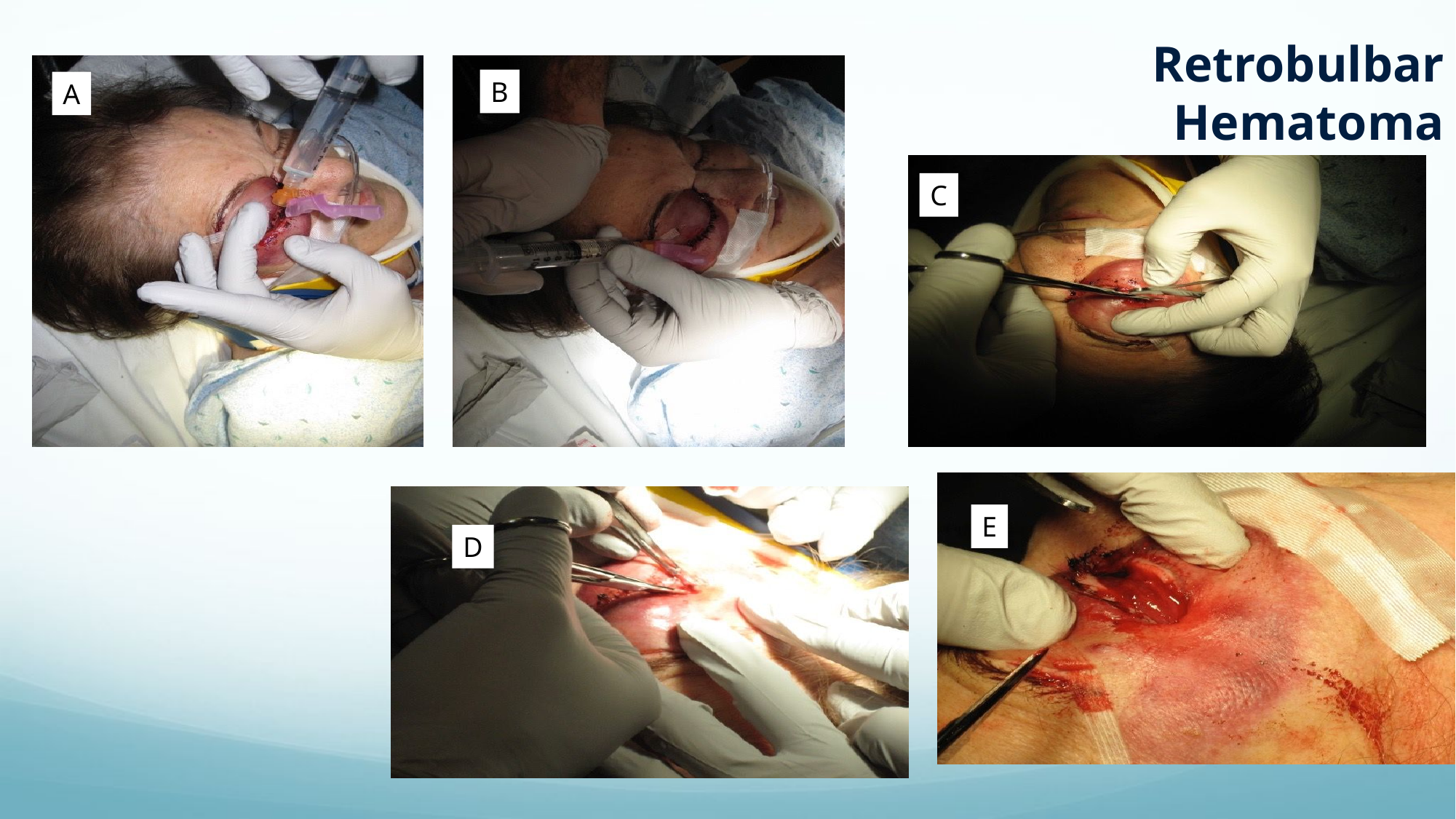

# Retrobulbar Hematoma
B
A
C
E
D

## Slide 12
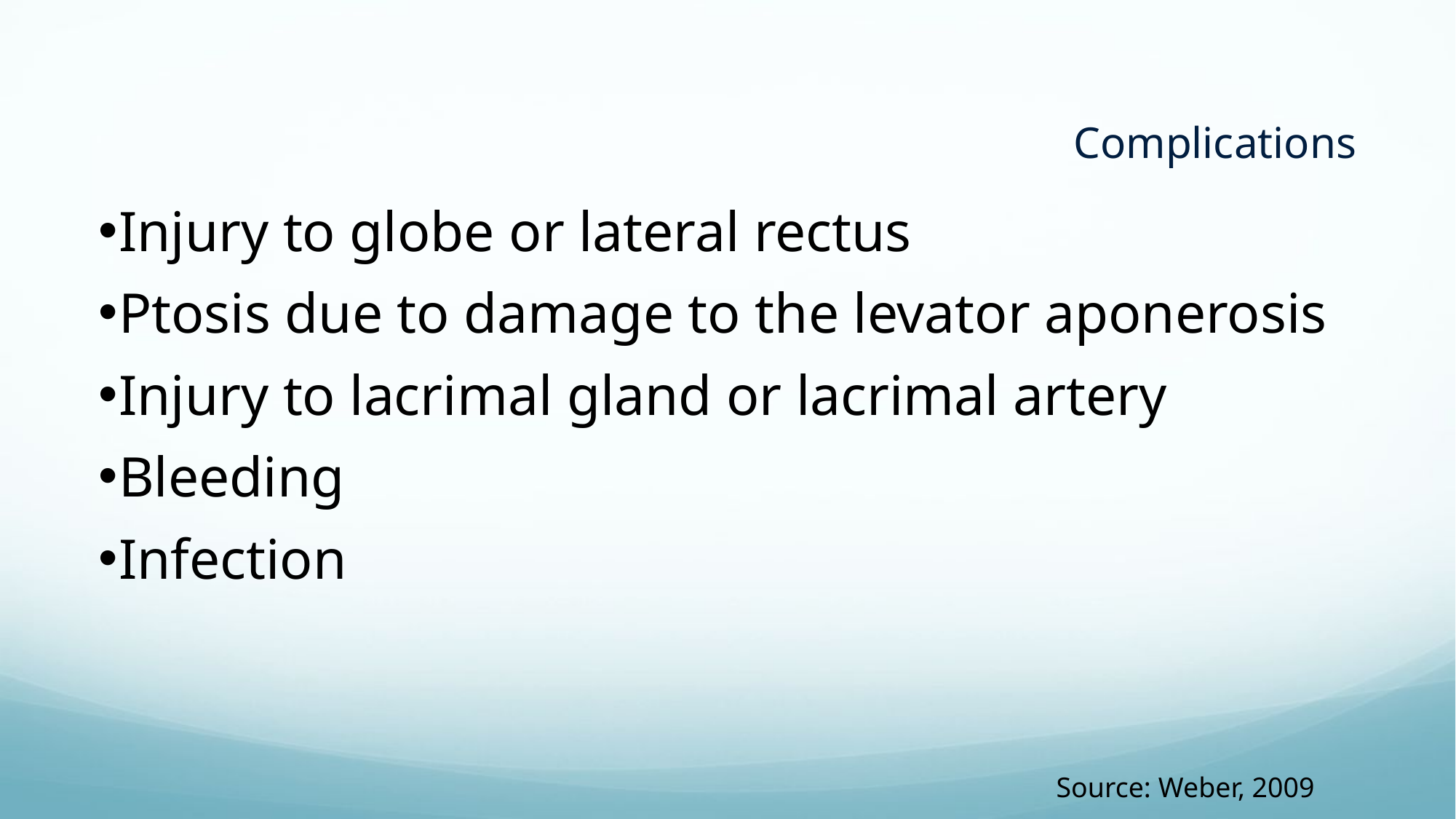

# Complications
Injury to globe or lateral rectus
Ptosis due to damage to the levator aponerosis
Injury to lacrimal gland or lacrimal artery
Bleeding
Infection
Source: Weber, 2009

## Slide 13
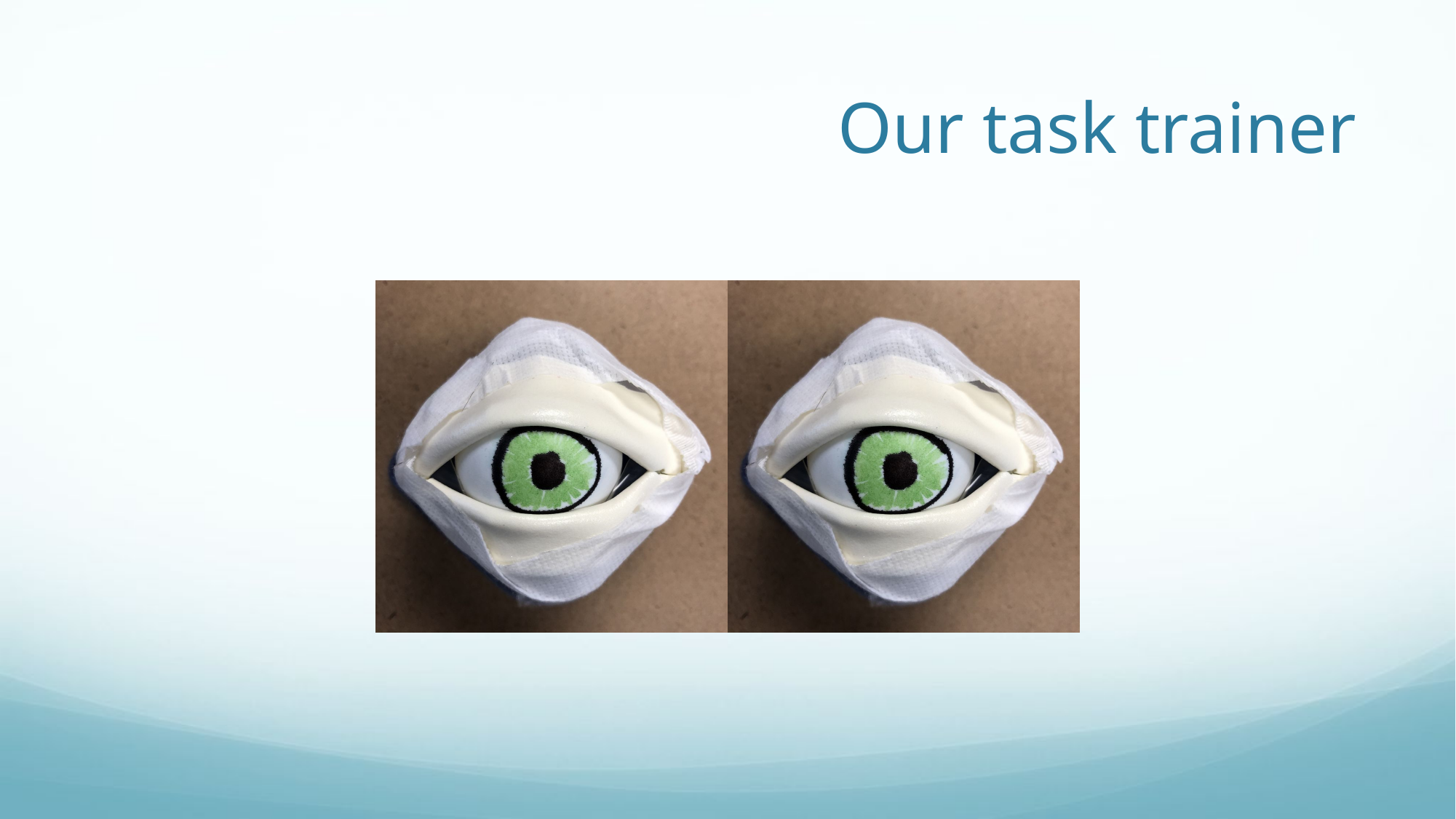

# Our task trainer

## Slide 14
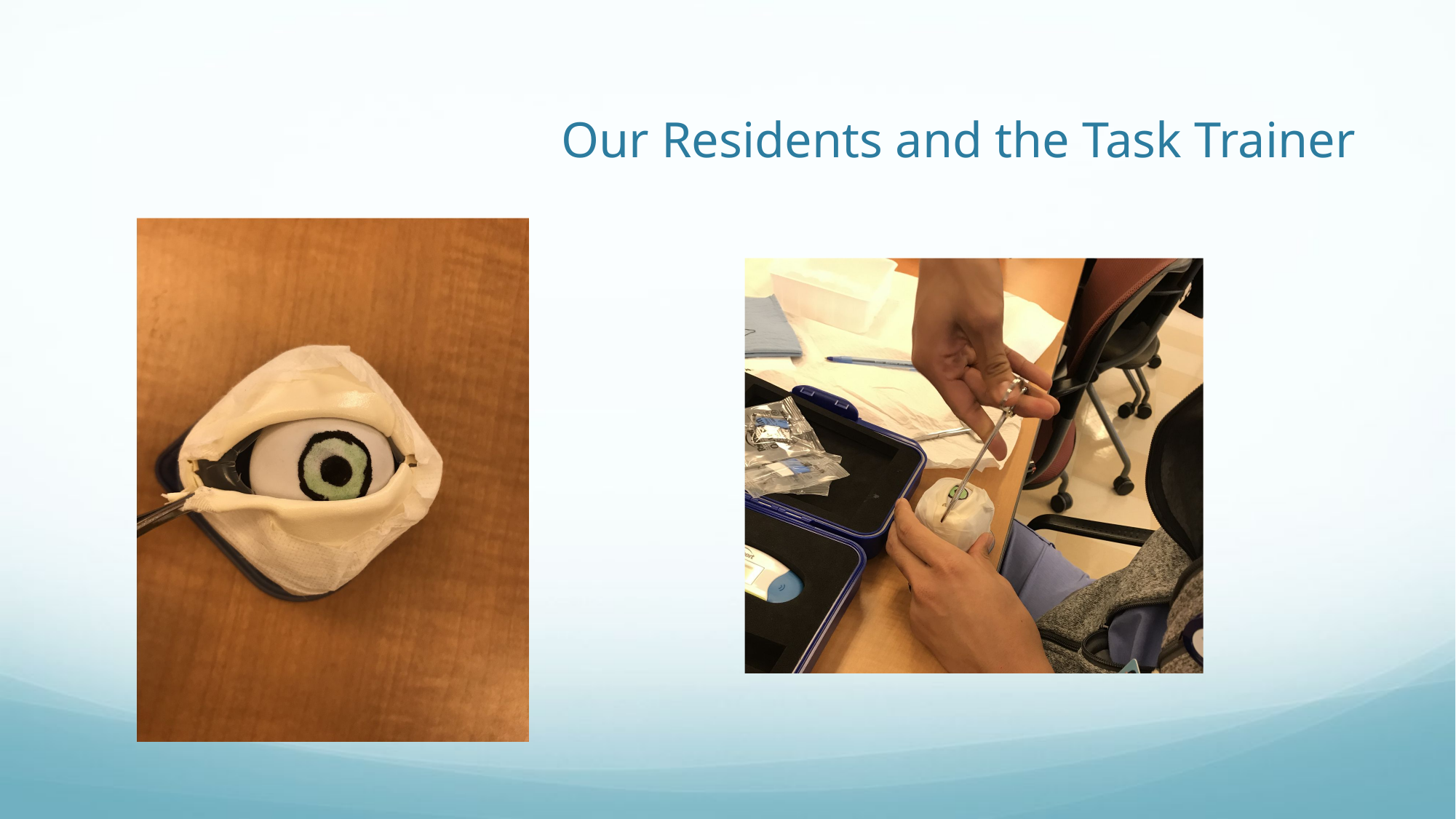

# Our Residents and the Task Trainer

## Slide 15
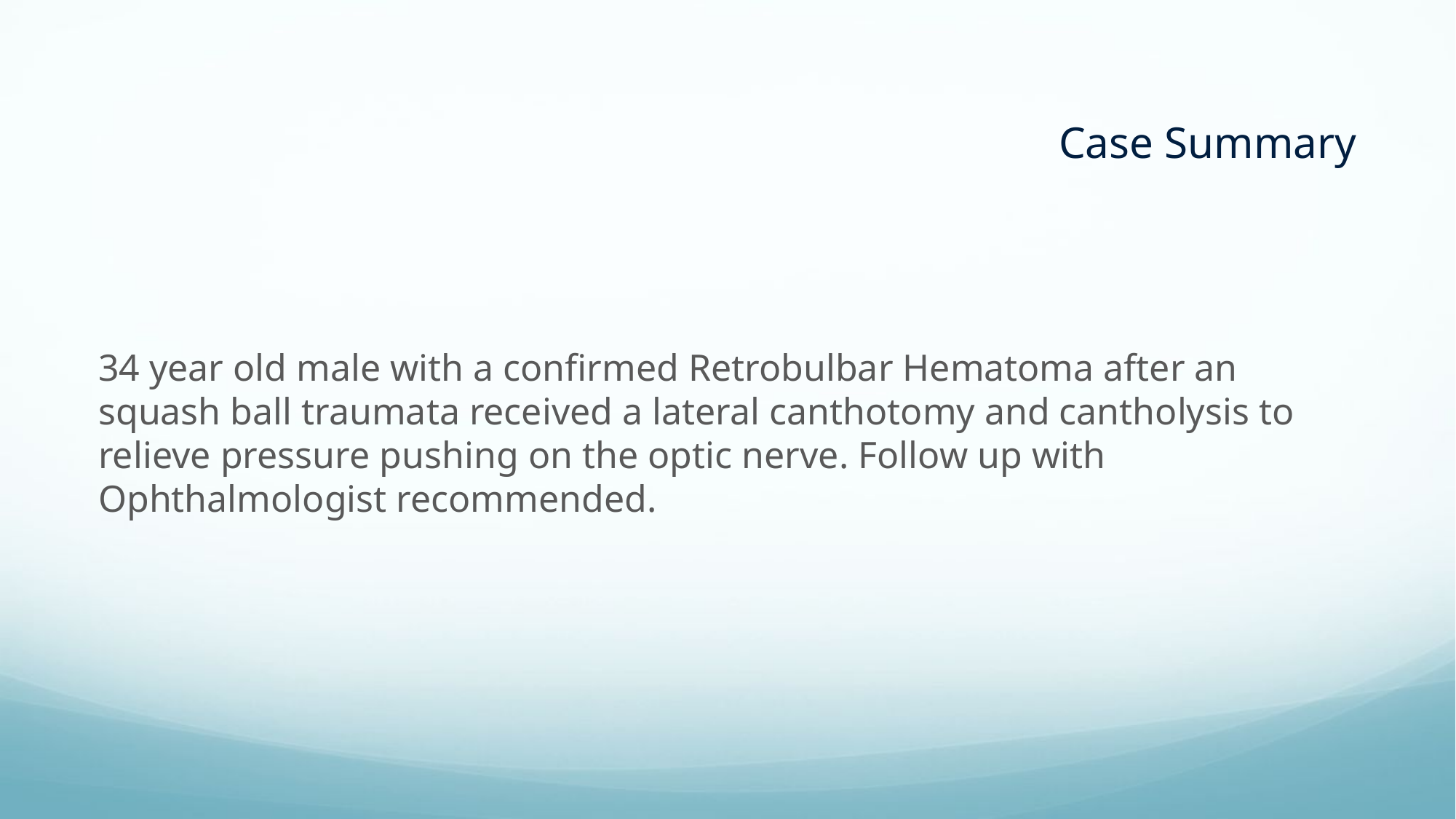

# Case Summary
34 year old male with a confirmed Retrobulbar Hematoma after an squash ball traumata received a lateral canthotomy and cantholysis to relieve pressure pushing on the optic nerve. Follow up with Ophthalmologist recommended.

## Slide 16
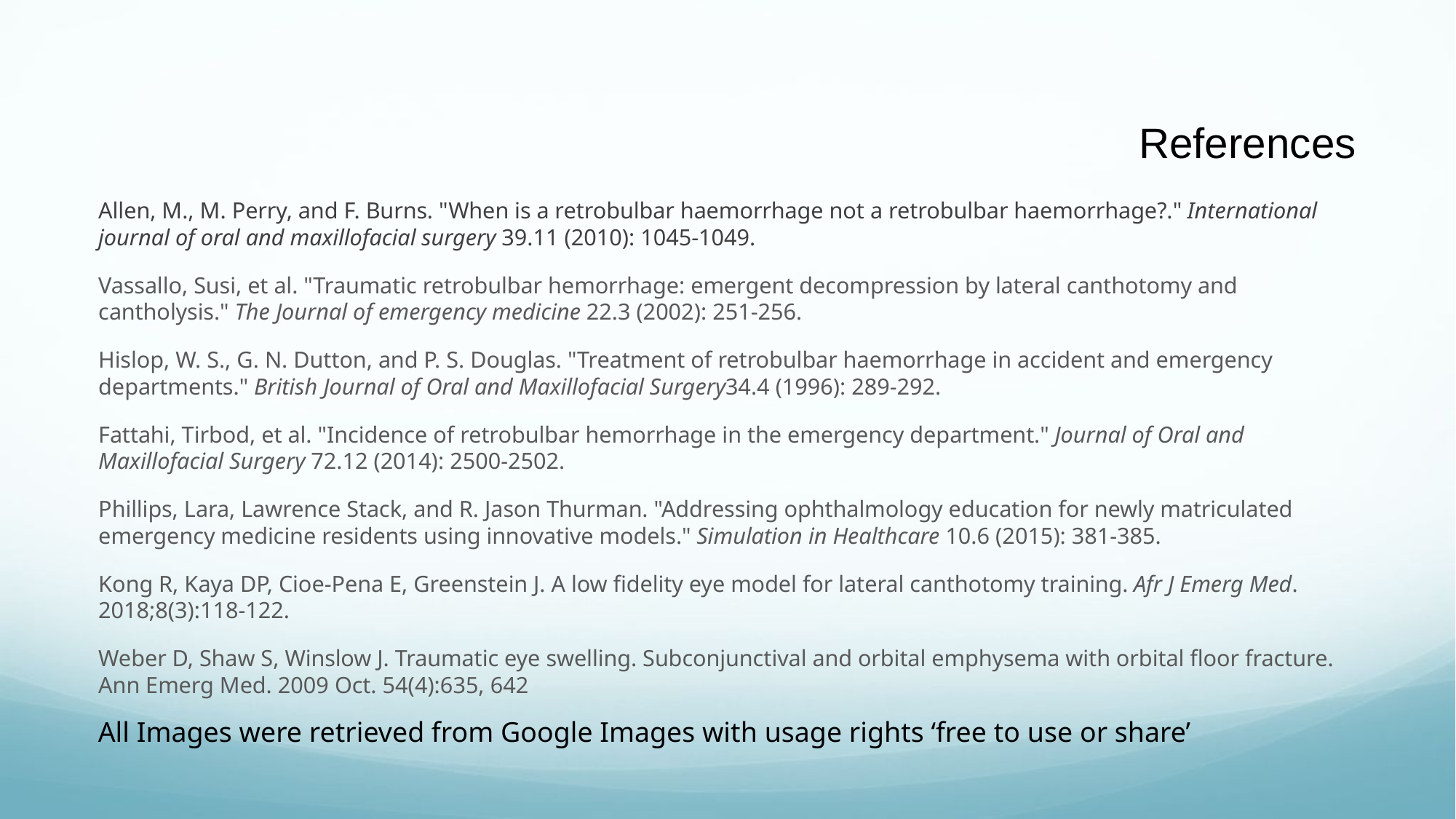

# References
Allen, M., M. Perry, and F. Burns. "When is a retrobulbar haemorrhage not a retrobulbar haemorrhage?." International journal of oral and maxillofacial surgery 39.11 (2010): 1045-1049.
Vassallo, Susi, et al. "Traumatic retrobulbar hemorrhage: emergent decompression by lateral canthotomy and cantholysis." The Journal of emergency medicine 22.3 (2002): 251-256.
Hislop, W. S., G. N. Dutton, and P. S. Douglas. "Treatment of retrobulbar haemorrhage in accident and emergency departments." British Journal of Oral and Maxillofacial Surgery34.4 (1996): 289-292.
Fattahi, Tirbod, et al. "Incidence of retrobulbar hemorrhage in the emergency department." Journal of Oral and Maxillofacial Surgery 72.12 (2014): 2500-2502.
Phillips, Lara, Lawrence Stack, and R. Jason Thurman. "Addressing ophthalmology education for newly matriculated emergency medicine residents using innovative models." Simulation in Healthcare 10.6 (2015): 381-385.
Kong R, Kaya DP, Cioe-Pena E, Greenstein J. A low fidelity eye model for lateral canthotomy training. Afr J Emerg Med. 2018;8(3):118-122.
Weber D, Shaw S, Winslow J. Traumatic eye swelling. Subconjunctival and orbital emphysema with orbital floor fracture. Ann Emerg Med. 2009 Oct. 54(4):635, 642
All Images were retrieved from Google Images with usage rights ‘free to use or share’
