# Supplementary material for: “Trauma to the Eye”—A Low Fidelity Resident Teaching Module for Identifying and Treating a Retrobulbar Hematoma
Source: MedEdPORTAL. 2021 Jan 25;17:11075. doi: 10.15766/mep_2374-8265.11075 (PMC7837065; doi:10.15766/mep_2374-8265.11075)
Supplement: Supplementary file 1 — Model Construction.docxAssessment Questionnaire.docxRH Checklist.docxCase and Supplemental Images.pptxSimulation Case Template.docx [file mep_2374-8265.11075-s001.zip › E. Simulation Case Template.docx]

| **Appendix E: MedEdPORTAL Simulation Case Template**  **SIMULATION CASE TITLE:** Trauma to the Eye  **AUTHORS**: Jared Raikin, BS,^1^ Ronald V. Hall, MD,^2^ Dimitrios Papanagnou, MD, MPH, RDMS, RDCS, CHSE^3^  **LEARNER AUDIENCE:** Emergency Medicine Residents | |
| --- | --- |
| **PATIENT NAME:** Casper MacQueen  **PATIENT AGE:** 34  **CHIEF COMPLAINT:** “pushing feeling behind my eye”  **PHYSICAL SETTING:** Emergency Room | |
|  | |
| **Brief narrative description of case** | Casper MacQueen, a 34-year-old man is brought to the ED by ambulance after being hit in the eye with a squash ball 30 minutes prior. Mr. MacQueen is alert and awake and arrived via ambulance with no family present. He says that he was playing squash with one of his friends and had forgotten his goggles at home. He explains that he lost sight of the ball at it hit him directly in the eye. He noticed a ‘pushing’ feeling behind his left eye, slight blurring of vision, and decided he needed to call an ambulance. |
| **Primary Learning Objectives** | 1. Diagnose a retrobulbar hematoma  2. Measure intraocular pressures  3. Identify relevant anatomical structures of the eye  4. Perform lateral canthotomy and cantholysis confidently  5. Describe appropriate follow-up care for a retrobulbar hematoma |
| **Critical Actions** | Measure IOP using Tonopen  Provide analgesia  Perform a LCC  Schedule proper follow-up care |
| **Learner Preparation or Prework** | No prework or preparation needed |

| Initial Presentation | | | | | |  |
| --- | --- | --- | --- | --- | --- | --- |
| **Initial vital signs** | Temp 98.1°F (37.2°C); HR 90/min; RR 14/min; BP 112/84 mm Hg; O2 sat 98% | | | | |  |
| **Overall Setting and Appearance** | Alert, anxious appearing male in moderate distress with severe contusion and moderate swelling around eye. 10 minutes after arrival in the ED the swelling around the eye has begun to increase and his left eye begins to swell shut. *Show picture from Appendix 2 | | | | |  |
| **Confederates (e.g., standardized participants) and their roles in the room at case start** | Facilitator can run alone, no confederates needed. | | | | |  |
| **HPI** | Casper was brought to the ED by ambulance after being hit in the eye with a squash ball 30 minutes prior. He is alert and awake and arrived via ambulance with no family present. He says that he was playing squash with one of his friends and had forgotten his goggles at home. He explains that he lost sight of the ball at it hit him directly in the eye. He noticed a ‘pushing’ feeling behind his left eye, slight blurring of vision, and decided he needed to call an ambulance. | | | | |  |
| **Past Medical/Surgical History** | **Medications** | | **Allergies** | | **Family History** |  |
| No Medical history  Appendectomy at 17 y.o. | Allegra for seasonal allergies | | None | | Family history is significant with father who died of a ‘heart attack’ at age 73 and a mother with breast cancer. |  |
| **Physical Examination** | | | | | |  |
| **General** | Alert, anxious appearing male in moderate distress | | | | |  |
| **HEENT** | Severe contusion around left eye, significant intraocular pressure (IOP) of left eye when palpated, moderate swelling around eye. | | | | |  |
| **Neck** | Supple | | | | |  |
| **Lungs** | Clear to P & A | | | | |  |
| **Cardiovascular** | RR no murmurs | | | | |  |
| **Abdomen** | Soft, no guarding or rigidity, bowel sounds active, Liver not palpable; spleen not palpable | | | | |  |
| **Skin** | Anicteric | | | | |  |
| **GU** | Normal bowel sounds | | | | |  |
| Instructor Notes - Changes and CASE Branch Points  Information for Facilitator: Patient is suffering from a RH due to the traumatic injury to his eye. This is a time sensitive injury that must be treated with a LCC within the first 2 hours of injury. If not done in time, permanent blindness may occur from compression of the optic nerve. Participants must be able to rule out various other injuries that could have occurred including globe rupture, which is contraindicated for LCC. This injury can be confirmed with a measurement of >40mmHg using a Tonopen. Participants should then inject an analgesic, crush the lateral canthus, cut the relevant tendons, and then re-measure pressure to confirm the procedures success with pressure release. | | | | | | |
| **Intervention / Time point** | | **Change in Case** | | **Additional Information** | | |
| Participant attempts to visually inspect the eye | |  | | Facilitator describes appearance as proptotic, edematous, with ecchymosis surrounding eye. | | |
| Participant tries to obtain visual acuity | |  | | Visual acuity is unremarkable | | |
| Participant measures IOP | |  | | IOP is 42mmHg | | |
| 7 minutes into the case | | Patients IOP begins to increase and their eye swelling increases | | IOP will improve if LCC is performed | | |

Ideal Scenario Flow

The learners enter the room and are told that a patient has arrived in the ER after trauma to the eye. One learner is asked to take lead on the case and begin to take a history and find out that the patient was hit in the orbit with a small squash ball and is now feeling pressure behind the eye. Other learners are encouraged to participate by asking more questions to rule out other diagnoses. Learners may be called on to answer specific questions like ‘what tests do you want to order’ and ‘what should we do next’ to encourage more learner engagement. Learners then rule out other injuries including a globe fracture and begin the treatment protocol for treating a RH. The facilitator should identify gaps in the learners’ knowledge throughout the case by presenting the information provided in the ‘instructor notes’ section as questions. This will allow the learners an opportunity to answer, and give an opportunity to didactically educate the learners to fill in gaps of knowledge. All the learners then have an opportunity to practice performing the steps of a LCC with the eye models provided using the checklist as a reference.

**Anticipated Management Mistakes**

1. Difficulty with using Tonopen: We found when using this case with EM residents that many of our learners did not know how to properly use a Tonopen. We modified our sessions to include a more detailed instruction and demonstration of how to properly use it.
2. Uncertainty in the type of injury that causes RH: Many of our learners were unsure about the types of injuries that more commonly result in a RH. We discussed the differential for injuries to highlight that a RH occurs from a direct blow to the orbit, and is less common with a larger area facial trauma.
3. Failure to recognize need for immediate treatment: Some of our learners did not understand the urgency of this injury. We modified our sessions to include a decline in patient status in the case after a short period of time to emphasize the important of treating a patient with a RH promptly.
